# Genome-Wide Mapping of Loci Associated With Resistance to Clubroot in *Brassica napus* ssp. *napobrassica* (Rutabaga) Accessions From Nordic Countries

**DOI:** 10.3389/fpls.2020.00742

**Published:** 2020-06-12

**Authors:** Rudolph Fredua-Agyeman, Zhiyu Yu, Sheau-Fang Hwang, Stephen E. Strelkov

**Affiliations:** Department of Agricultural, Food and Nutritional Science, University of Alberta, Edmonton, AB, Canada

**Keywords:** rutabaga, marker-clubroot association, *Plasmodiophora brassicae*, pathotypes, genomic hotspot

## Abstract

Rutabaga [*Brassica napus* ssp. *napobrassica* (L.) Hanelt] is reported to be an excellent source of clubroot (*Plasmodiophora brassicae*) resistance genes. In this study, 124 rutabaga accessions from the Nordic countries (Norway, Sweden, Finland, Denmark, and Iceland) were evaluated for their reaction to five single-spore isolates representing *P. brassicae* pathotypes 2F, 3H, 5I, 6M, and 8N and 12 field isolates representing pathotypes 2B, 3A, 3O, 5C, 5G, 5K, 5L, 5X (two isolates, L-G2 and L-G3), 8E, 8J, and 8P. The accessions were also genotyped using a 15K Brassica SNP array and 60 PCR-based primers linked to previously identified clubroot resistance genes. Six thousand eight hundred sixty-one SNP markers were retained after filtering with TASSEL 5.0, and used to evaluate four general linear models (GLM) and four mixed linear models (MLM). The PCA + K and Q + K MLM models gave the minimal deviance of the observed from the expected distribution in quantile-quantile plots, and hence were used for SNP-clubroot association analyses. In addition, 108 alleles derived from the PCR-based markers and the phenotypic data were analyzed with the PCA + K model. Forty-five SNPs and four PCR-based markers were identified to be associated strongly with resistance to isolates representing 13 pathotypes (2F, 3H, 5I, 6M, 8N, 2B, 3A, 3O, 5C, 5G, 5K, 5L, and 8P). These markers revealed the top and bottom segments of rutabaga chromosome A03 and the middle segment of chromosome A08 as genomic hotspots associated with resistance to the different *P. brassicae* pathotypes.

## Introduction

Rutabaga (*Brassica napus* ssp. *napobrassica*) has been cultivated commercially as a vegetable crop in Canada since the 1950s and 1960s (Shattuck and Proudfoot, 1990; Spaner, 2002). This crop is affected by a number of diseases including clubroot of crucifers, caused by the obligate parasite *Plasmodiophora brassicae*. Clubroot is best managed by the use of host resistance (Rahman et al., 2014). The European winter oilseed rape (syn. canola; *B. napus*) ‘Mendel’ has been the main source of clubroot resistance gene(s) in many canola breeding programs in Canada (Fredua-Agyeman et al., 2018), but ‘new’ pathotypes of *P. brassicae* capable of overcoming this resistance in clubroot resistant (CR) canola cultivars have emerged recently in Alberta (Strelkov et al., 2016, 2018). Indeed, the *CRa*/*CRb*^Kato^ resistance gene(s) on the A03 chromosome of ‘Mendel’ (Fredua-Agyeman and Rahman, 2016) were found to confer resistance to only about 50% of the new *P. brassicae* pathotypes identified in Alberta from 2013 to 2015 (Fredua-Agyeman et al., 2018). As such, efforts have intensified to identify new clubroot resistance genes from other Brassica sources to develop the next generation of CR canola cultivars.

Greenhouse resistance screening showed that rutabagas possess both qualitative and quantitative resistance to isolates representing the old and new pathotypes of *P. brassicae* (Hasan et al., 2012; Fredua-Agyeman et al., 2019). Therefore, rutabagas can be used in the breeding of CR canola cultivars. Examples of CR rutabaga cultivars developed in Canada include ‘Chignecto,’ ‘York,’ ‘Fortune,’ ‘Kingston,’ and ‘Brookfield’ (Shattuck and Proudfoot, 1990). The Canola Breeding Program at the University of Alberta mapped the *Crr1* clubroot resistance gene in ‘Brookfield’ to the A08 chromosome of *B. rapa* and *B. napus* (Hasan and Rahman, 2016). While rutabaga is a root vegetable and canola is an oilseed, they are both *B*. *napus* and share the same ploidy level and genome (2*n* = 38, AACC). Therefore, the introgression of clubroot resistance genes from rutabaga to canola does not face many of the pre- and post-zygotic challenges associated with crosses with more distant relatives. On the other hand, many cycles of backcrossing and breeding will be needed to achieve canola quality (spring growth characteristics, plant architecture, reductions in the high content of erucic acid in the oil and glucosinolate in the seed meal, as well as days to flower). In addition, molecular markers linked to the clubroot resistance genes in rutabagas need to be identified for marker-assisted selection (MAS) to track the introgressed genes.

In plants, linkage-based mapping is often carried out on populations developed from bi-parental crosses, and so polymorphism is restricted to the contrasting genetic variability for the trait of interest in the parents (Brachi et al., 2011; Gupta et al., 2014). As such, markers detected for quantitative traits are restricted to the two parents and may not work in populations developed from other parents. An advantage of linkage-based QTL mapping is the often very high power of QTL detection (Gupta et al., 2014). Linkage disequilibrium (LD)-based association mapping (AM), such as genome-wide association mapping (GWAS), is an alternate approach for capturing recombination events to the gene level in natural populations, and has several advantages over traditional linkage-based mapping (Gupta et al., 2014). GWAS can be applied to a diverse set of genotypes of any crop species, does not require ancestry or pedigree information for QTL mapping, captures higher allelic diversity, provides higher resolution, can be used to study many traits of interest, and is cheaper and faster to complete since it does not require the development of a mapping population. However, LD-based AM is often limited by the detection of false positive associations (Type I error) that may arise as the result of linkage, population stratification and relatedness among individuals (Gupta et al., 2014). In addition, SNPs with minor alleles (<5–10%) are filtered off during GWAS analyses, and hence GWAS lacks the power to detect these minor alleles (Brachi et al., 2011).

The objectives of this study were to phenotype and genotype rutabaga accessions collected from Norway, Sweden, Finland, Denmark, and Iceland, and to use GWAS to identify SNP and SSR markers significantly associated with clubroot resistance to a collection of *P. brassicae* isolates from Alberta, Canada, representing different pathotypes. In addition, the positions of the identified markers, as well as those from previous genetic mapping studies, were traced to the *B*. *rapa*, *B. oleracea*, and *B. napus* reference genomes. Lastly, candidate genes associated with the identified genomic regions were identified based on the proteins they encode.

## Materials and Methods

### Plant Material

The materials used in this study comprised all 124 *B. napus* ssp. *napobrassica* (rutabaga) accessions used for the genetic diversity studies of Yu (2020). Population-genetic differences were captured in the aforementioned study with the software *STRUCTURE* v2.3.4 (Pritchard et al., 2000). Seeds of the rutabaga were multiplied by planting 2–4 seeds of each accession in 13 × 13 × 15 cm pots filled with Sunshine Mix #4 Aggregate Plus growing mix (Sungro Horticulture, Seba Beach, Alberta, Canada), and placing the pots in a greenhouse at the Crop Diversification Centre North (CDCN), Alberta Agriculture and Forestry (AAF), Edmonton, Canada. After 1 week, the seedlings were thinned to two plants per pot and kept in the greenhouse for another 5–6 weeks at 20–25^o^C/15–18^o^C day/night temperatures and a 16 h light/8 h dark photoperiod. The plants were then vernalized for 12 weeks in a cold room maintained at 4^o^C with a 12 h photoperiod, and subsequently returned to the greenhouse for flowering, maturation and seed harvest.

### *Plasmodiophora brassicae* Pathotypes

The *P. brassicae* pathotypes used in the clubroot tests comprised 12 of the field isolates reported by Strelkov et al. (2016, 2018) to cause elevated disease on clubroot resistant (CR) canola and five single-spore isolates (SSIs) identified by Xue et al. (2008) prior to the introduction of CR canola to western Canada. The 12 virulent isolates (L-G2 + L-G3, D-G3, F183-14, F3-14, F175-14, CDCN#6, F187-14, F10-15, F12-15, F381-16/C.C. and UofA/County#37) were classified according to the Canadian Clubroot Differential (CCD) Set as pathotypes 5X, 5L, 2B, 3A, 5C, 5G, 8E, 5K, 8J, 3O, and 8P, respectively (Strelkov et al., 2018). Two of the field populations L-G2 and L-G3 (Strelkov et al., 2016) represented the same pathotype (5X). Therefore, the 12 virulent isolates represented 11 pathotypes.

The five SSIs were classified as pathotypes 2, 3, 5, 6, and 8 on the differentials of Williams (1966) and as pathotypes 2F, 3H, 5I, 6M, and 8N, respectively on the CCD Set (Strelkov et al., 2018). The numbers in the CCD designations correspond to the classification according to the differentials of Williams, while the letters designate the unique virulence patterns on the hosts of the CCD. The 17 isolates (representing isolates of 16 pathotypes) used in this study were maintained as galls on the universally susceptible European Clubroot Differential (ECD) 05 (Buczacki et al., 1975) at −20^o^C.

### Clubroot Phenotyping

Greenhouse clubroot tests were conducted at the CDCN following Fredua-Agyeman et al. (2019). Briefly, 4–8 seeds of each rutabaga accession were placed on Whatman No. 1 filter paper wetted with distilled water in Petri dishes, and kept at room temperature (18–22^o^C) and a 12 h light/12 h dark photoperiod provided by white fluorescent light. The filter paper was moistened daily with water for 7 days, at which point the seeds had germinated. The universally susceptible *B. napus* (canola) cv. ‘Westar’ was included in each experiment as a positive control.

Inoculum of isolates representing each *P. brassicae* pathotype (2F, 3H, 5I, 6M, 8N, 5X (L-G2 and L-G3), 5L, 2B, 3A, 5C, 5G, 8E, 5K, 8J, 3O, and 8P) was prepared by macerating the frozen galls with a Waring LB10G variable-speed blender (Cole-Palmer) and filtering the resulting homogenate through two layers of cheesecloth. The resting spore concentration in the filtrate was measured with a hemocytometer and adjusted to 1.0 × 10^7^ resting spores mL^–1^ with water. Seven-day old seedlings of each of the 124 rutabaga accessions were inoculated by dipping the roots in the spore suspension as described by Nieuwhof and Wiering (1961).

The inoculated seedlings were transplanted into 13 cm × 13 cm × 15 cm pots filled with Sunshine Mix #4 Aggregate Plus soilless mix (Sungro Horticulture Canada Ltd., Seba Beach, Canada), followed by the addition of 1 mL of inoculum into the potting mix at the base of each plant (Lamers and Toxopeus, 1977 as cited by Voorrips and Visser, 1993). The inoculated rutabaga seedlings were planted at the periphery of each pot while one seedling of the susceptible check ‘Westar’ was placed at the center. Pots inoculated with the same isolate were placed randomly on one bench and re-randomized twice on the 3rd and 6th weeks after transplanting.

The experiments were repeated 3–4 times based on seed availability. Maintenance of the plants (watering, fertilizing and insect pest control) and growing conditions (photoperiod and temperature) in the greenhouse were as described by Fredua-Agyeman et al. (2019).

### Clubroot Disease Rating and Phenotypic Variation

The severity of clubroot was assessed 8 weeks after inoculation on a 0–3 scale (Kuginuki et al., 1999) where: 0 = no galling, 1 = a few small galls on the lateral roots, 2 = moderate galling on the lateral roots but not on the main root, and 3 = severe galling on both the lateral and main root. Ratings for the rutabaga accessions in each pot were considered valid only if the disease rating of the susceptible check ‘Westar’ in the same pot was 2 or 3. An index of disease (ID, 0–100%) was calculated for each isolate/host genotype combination following Strelkov et al. (2006), with the mean IDs and standard deviations (SDs) of the repeated experiments determined for all 124 rutabaga accessions for each of the 17 *P. brassicae* isolates.

The rutabaga accessions were designated as resistant (Mean ID + SD ≤ 30%), moderately resistant (30% < Mean ID + SD ≤ 50%) or susceptible (Mean ID + SD > 50%) to isolates representing each pathotype based on Fredua-Agyeman et al. (2019) and the clubroot resistance screening guidelines of the Western Canada Canola/Rapeseed Recommending Committee (WCC/RCC). The grand mean (GM) IDs (means of the means of IDs with all 17 isolates) of each accession were also calculated and used as an indicator of broad-spectrum resistance. The percentage of the accessions that were resistant (R), moderately resistant (MR), or susceptible (S) were calculated. In addition, the frequency distribution of the resistance reaction of the rutabaga accessions to each pathotype was tested for normality. PROC TRANSREG (Box-Cox and Log transformation) in SAS v. 9.4 (SAS Institute, United States) and the rank based inverse normal transformation (INT) were carried out on the phenotypic data that did not yield normal curves.

### DNA Extraction

DNA was extracted from ca. 0.25 g leaf tissue of each accession using the cetyltrimethyl ammonium bromide (CTAB) method (Sambrook and Russell, 2001). The DNA concentration was measured with a ND-2000C spectrophotometer (NanoDrop, Technologies, Inc., Wilmington, DE, United States) and the concentration diluted to 10–20 ng μL^–1^ for the working solution of each DNA sample.

### SNP Genotyping

SNP genotyping was performed using the *Brassica* 15K SNP array at TraitGenetics GmbH, Gatersleben, Germany, according to the manufacturer’s protocols (Clarke et al., 2016). After removing monomorphic, low coverage site markers, markers with MAF ≤ 0.05 and those missing data for >5% of the accessions, 6861 SNP markers, comprising 4390 A-genome and 2471 C- genome markers, were used for GWAS analyses.

### PCR and Genotyping With PCR-Based Markers Linked to Known CR Genes

PCR reactions, genotyping, amplified product detection and calling of allele sizes were performed as described by Fredua-Agyeman et al. (2014).

The genotyping was carried out with 60 PCR-based primers linked to seven previously identified clubroot resistance genes. Primers screened from the A03 chromosome of *B. rapa* consisted of the following: six SCAR markers linked to the *CRa* gene (Ueno et al., 2012), three SSR, four SCAR and one CAP marker(s) linked to the *CRb* gene (Piao et al., 2004; Zhang et al., 2014), 32 SSR and three InDel markers linked to the *CRb*^Kato^ gene (Kato et al., 2012, 2013), one STS marker linked to the *Crr3* gene (Saito et al., 2006), one CAP marker linked to the *Rcr1* gene (Chu et al., 2014), one SSR and one SCAR marker linked to the *CRk* gene (Suwabe et al., 2006; Matsumoto et al., 2012) and two SSR markers linked to the *CRd* gene (Pang et al., 2018).

Primers screened from other chromosomes comprised: three SSR markers on the A08 chromosome of *B. rapa* linked to the *Crr1* gene (Suwabe et al., 2006; Hasan and Rahman, 2016; Hobson and Rahman, 2016), one SSR marker on the A01 chromosome linked to the *Crr2* gene and one SSR marker on the A06 chromosome linked to the *Crr4* gene (Suwabe et al., 2003, 2006). The primer names, sequences and chromosomal locations are listed in Supplementary Table S1.

Each allele was coded as a separate site with each allele in turn recoded as one of two nucleotides (A or T; C or G) as specified in the TASSEL Manual (Bradbury et al., 2007). Genotyping was repeated for 10% of the individual samples to confirm the reproducibility of the molecular data (Fredua-Agyeman et al., 2014). In addition, alleles with a frequency of ≤5% were removed to reduce false positive associations (Nie et al., 2016). One hundred and eight alleles linked to known CR genes were used for the GWAS analyses.

### Linkage Disequilibrium

One hundred and ten more distant SNP markers which unduly increased the length of SNP coverage on 10 chromosomes were removed before LD analysis. These comprised 6, 13, 8, 28, 41, 2, 2, 1, 2, and 7 SNPs from chromosomes A04, C01, C02, C03, C04, C05, C06, C07, C08, and C09, respectively. Therefore, a total of 6751 (4384 A-genome + 2367 C-genome) uniformly distributed SNPs were used to determine the extent of LD.

Intra-chromosomal linkage disequilibrium in the rutabaga accessions was analyzed by calculating the squared value of the correlation coefficient of the allele frequencies (r^2^) between all pairs of linked loci with TASSEL 5.0 (Bradbury et al., 2007). However, due to computational challenges, the inter chromosomal pairwise comparison of *r*^2^-values could not be obtained directly for the 4384 SNP markers (9607536 pairs) on chromosomes A_1_–A_10_, the 2367 SNP markers (2800161 pairs) on chromosomes C_1_-C_9_ and all 6751 SNP markers (22784625 pairs) for the entire genome. Instead, the data of the pairwise comparison of linked SNP markers on each chromosome were used to obtain the mean values for the A- and C- genomes, as well as for the entire AC genome of rutabaga (Table 1).

**TABLE 1:** SNP marker density and extent of intra-chromosomal linkage disequilibrium in rutabaga (*Brassica napus* ssp. *napobrassica*).

Linkage group or chromosome	Number of SNP markers	Length covered (kb)	Average inter-SNP marker distance (kb)	Pairwise comparisons of all linked SNP markers	Number (%) of SNP pairs in significant LD^ϕ^	Average *r*^2^-value/chromosome	Estimated LD decay (kb)^ψ^
A01	394	28231.6	71.6	77421	13644 (17.6)	0.039	1700
A02	344	27644.6	80.2	58996	12441 (21.1)	0.052	2000
A03	668	31540.9	47.2	222778	45474 (20.4)	0.039	1500
A04*	419	18619.9	44.4	87571	18213 (20.8)	0.045	1500
A05	375	26887.8	71.7	70125	15774 (22.5)	0.047	2300
A06	461	26159.7	56.8	106030	20147 (19.0)	0.037	1200
A07	679	22474.7	33.1	230181	47227 (20.5)	0.040	1100
A08	282	21417.2	75.9	39621	11100 (28.0)	0.055	1300
A09	348	37014.2	106.3	60378	9527 (15.8)	0.035	1900
A10	414	19137.5	46.1	85491	14448 (16.9)	0.038	1100
C01*	87	2104.2	24.2	3741	1204 (32.2)	0.068	520
C02*	303	3117.5	10.3	45753	12144 (26.5)	0.083	1500
C03*	443	2516.2	5.7	97903	22827 (23.3)	0.050	200
C04*	446	2419.4	5.4	99235	23434 (23.6)	0.051	240
C05*	168	2053.6	12.2	14028	2876 (20.5)	0.079	440
C06*	413	3210.1	7.8	85078	25320 (29.8)	0.073	280
C07*	219	4881.0	22.3	23871	4940 (20.7)	0.054	770
C08*	210	4374.5	20.8	21945	4101 (18.7)	0.059	560
C09*	78	2060.8	26.4	3003	764 (25.4)	0.076	440
A-genome	4384	259128.1	63.4 ± 21.9	1038592	207995 (20.3 ± 3.4)	0.041	1560.0 ± 408.8
C-genome	2367	26737.2	15.0 ± 8.4	394557	97610 (24.5 ± 4.4)	0.063	550.0 ± 398.1
AC-genome	6751	285865.3	40.5 ± 29.8	1433149	305605 (22.3 ± 4.4)	0.047	1081.6 ± 649.9

*^∗^Chromosomes which had more distant SNP markers removed before determination of extent of linkage disequilibrium. ^ϕ^The number and percentage of SNP pairs in significant LD were determined from Chi-squared tests at *p* < 0.001. ^ψ^The extent of LD decay was estimated from the projection of the intersection between the fitted curve of the data points and the 95th percentile of unlinked *r*^2^ threshold line (background LD) onto the physical distance axis.*

The significance of pairwise marker *r*^2^-values was determined by calculating the Chi-square (χ^2^) statistic for each SNP pair according to Zhou et al. (2012), except that a threshold *p*-value < 0.001 was used to assess the level of significance. The PROC GPLOT procedure in SAS v. 9.4 (SAS Institute) was used to generate LD plots of the *r*^2^-values of pairs of markers with *p* < 0.001 vs. physical map distance (in Mb) for each chromosome. The data points were then fitted with a solid curve using the PROC TRANSREG function in SAS. Background linkage disequilibrium (BLD) was calculated as the *r*^2^-values for unlinked markers that exceeded 95% (95th percentile) of the data set, following Breseghello and Sorrells (2006). The average extent of LD of each chromosome was estimated by the projection of the intersection between the fitted curve and the r^2^ threshold line onto the physical distance axis (Breseghello and Sorrells, 2006; Bellucci et al., 2015).

### Marker-Clubroot Association

To identify loci in the rutabaga accessions associated with the response to each of the 17 *P. brassicae* isolates, genome-wide association analyses were conducted with TASSEL 5.0 (Bradbury et al., 2007) using the 6861 SNP marker data and the mean ID values of each accession and pathotype. A separate analysis was performed using the 108 alleles derived from the 60 PCR-based markers and the mean ID data from the clubroot phenotyping experiments.

Marker-trait association analyses were carried out following Li et al. (2014) with slight modifications. Four models each were tested with the general linear models (GLM) and mixed linear models (MLM) procedures implemented in TASSEL 5.0 (Bradbury et al., 2007). The GLM tested comprised the N (Naïve), Q-only (population structure), K-only (Kinship) and PCA-only (Principal Component Analysis) models. The MLM tested comprised the Q + K and PCA + K models (Li et al., 2014) and two additional MLM association tests of Q and PCA using Distance matrices (D) (i.e., Q + D and PCA + D).

Quantile-Quantile (Q-Q) plots, which plot the –log_10_ *P*-value of the test of association (observed) with that expected given the null hypothesis of no marker-trait associations were obtained for each model. The results for the best GWAS model(s) were presented as Manhattan plots. Markers with strong associations to clubroot resistance were identified from the peaks on Manhattan plots. The Bonferroni correction was used to set the significance cut-off (α/*n*, where α = level of significance, *n* = number of markers) for both the SNP and the PCR-based markers. The SNP markers were considered to be significantly associated with the traits if *P* ≤ 1 × 10^–4^ (i.e., -log_10_ *P* = 4.0; 1/n, n = number of markers) based on the adjusted Bonferroni correction method (Benjamini and Hochberg, 1995). In the case of the PCR-based markers, a slightly lower threshold of *P* ≤ 5 × 10^–4^ (i.e., -log_10_ *P* = 3.3) was used to indicate the significance of associations between the markers and the traits.

The physical positions of the SNP and the SSR markers with strong associations to clubroot resistance were then mapped to the *B. rapa*, *B. oleracea*, and *B. napus* genome sequences to identify their association with previously characterized clubroot resistance genes.

### Candidate Gene Prediction

The sequences of the PCR-based and SNP markers found to be associated with clubroot resistance loci were used in BlastN searches of the *B. rapa* (AA), *B. oleracea* (CC), *B. napus* (AACC) and *Arabidopsis thaliana* genome sequences available in the NCBI^1^ GenBank database to determine their possible functions. An *E*-value ≤ E-20 and a percentage identity of ≥ 95% were used as the threshold cut-offs to confirm the putative functions of the candidate genes.

### Statistical Analyses

Statistical analyses of the phenotype data were conducted with SAS v. 9.4 (SAS Institute, United States). The PROC SORT function was used to select the R and MR (i.e., ID ± SD ≤ 50%) accessions. Duncan’s test (Steel and Torrie, 1960) was used to test (*P* ≤ 0.05) for differences among the ID mean values of all 17 isolates and to quantify these differences among the rutabaga accessions. The distribution of the IDs for each R and MR rutabaga accession were visualized with box-and-whisker plots.

## Results

### Phenotypic Variation of Clubroot Resistance in Rutabaga Accessions

**FIGURE 1:**
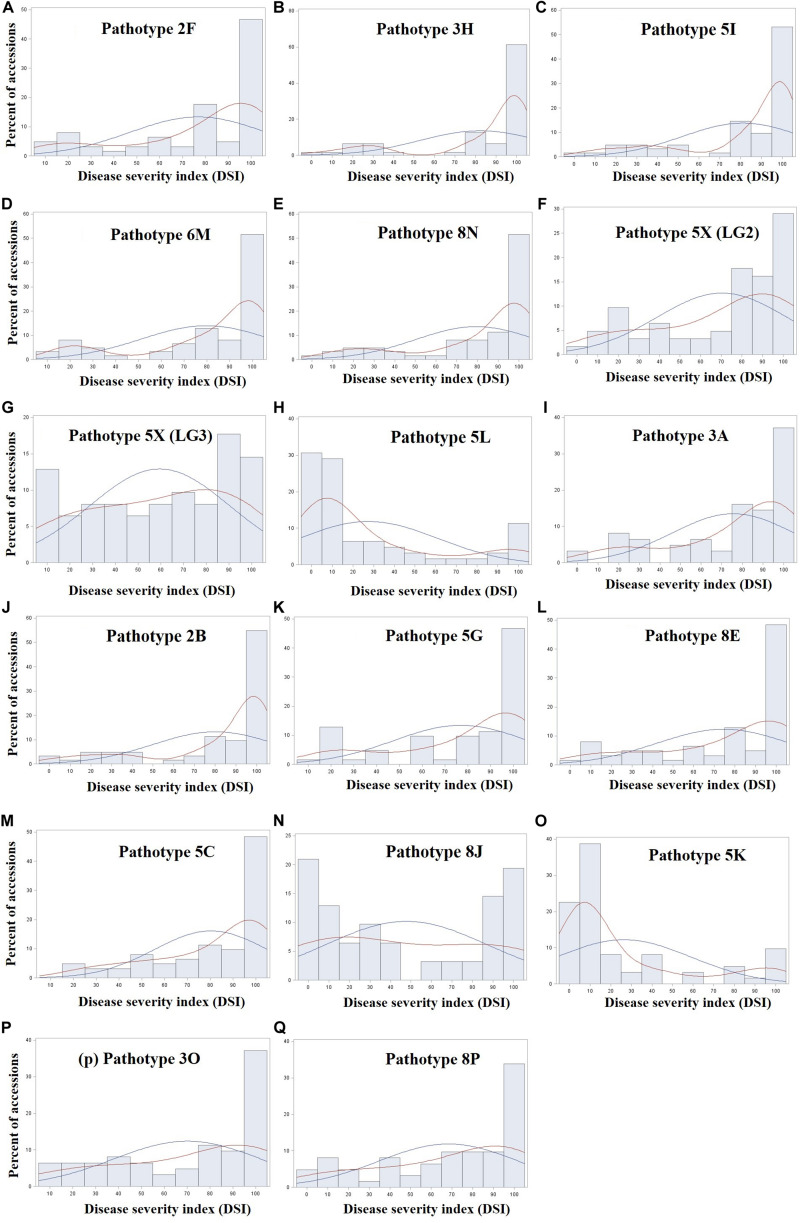
Frequency distribution of 124 rutabaga (*Brassica napus* ssp. *napobrassica*) accessions evaluated in greenhouse experiments for resistance to 17 *Plasmodiophora brassicae* isolates **(A–Q)** representing 16 different pathotypes. Pathotypes 2F, 3H, 5I, 6M, and 8N are single-spore isolates identified prior to the introduction of clubroot resistant (CR) varieties in Canada, while pathotypes 5X (LG2 and LG3), 5L, 3A, 2B, 5G, 8E, 5C, 8J, 5K, 3O, and 8P are represented by field isolates identified after the introduction of CR varieties in Canada. The blue and red fitted curves represent normal (parametric) and Kernel density (non-parametric) estimation of the distribution.

The frequency distribution of 124 rutabaga accessions evaluated for resistance to 17 *P. brassicae* isolates (representing 16 pathotypes) is shown in Figure 1. The phenotypic values for two of the 17 traits were bimodal distributed (Figures 1G,N), two (Figures 1H,O) were skewed to the right, while the remaining 13 distributions were skewed to the left (Figures 1A–F,I–M,P,Q). Unfortunately, transformation and rank-based inverse normal transformation of the data did not yield superior normal curves. The complexity of the disease, virulence of the isolates and the susceptibility of the host plants might have accounted for the skewness of the data even under transformation. The descriptive statistics for the reactions of the rutabaga accessions (Supplementary Table S2) to the different *P. brassicae* isolates suggested that, in spite of the largely skewed phenotypic data, sufficient resistant and susceptible resources existed to carry out this GWAS study.

**FIGURE 2:**
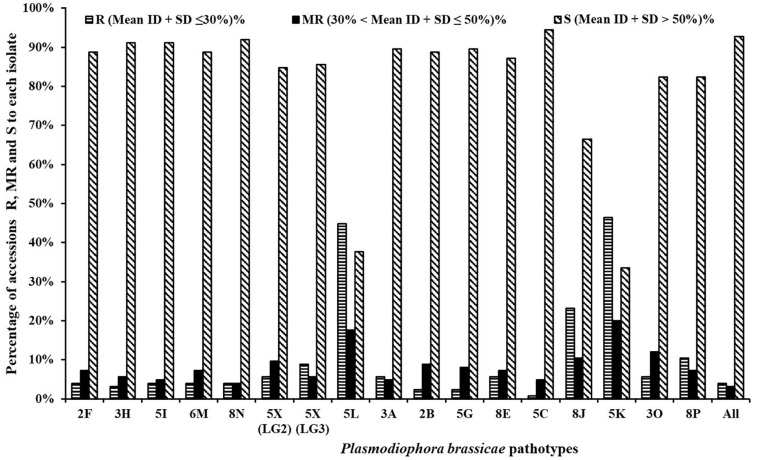
Frequency distribution for resistance resources in 124 rutabaga (*Brassica napus* ssp. *napobrassica*) accessions collected from Norway, Sweden, Finland, Denmark, and Norway. Pathotypes 2F, 3H, 5I, 6M, and 8N are single-spore isolates identified prior to the introduction of clubroot resistant (CR) varieties in Canada, while pathotypes 5X (LG2 and LG3), 5L, 3A, 2B, 5G, 8E, 5C, 8J, 5K, 3O, and 8P are represented by field isolates identified after the introduction of CR varieties in Canada.

Between 0.8 and 10.4% of the rutabaga accessions were resistant (R) and 4.0–12.0% were moderately resistant (MR) to 14 of the 17 *P. brassicae* isolates (Figure 2). The 14 isolates represented 13 pathotypes and this included all five SSIs (representing pathotypes 2F, 3H, 5I, 6M, and 8N) and 9 of the 12 field isolates [pathotypes 2B, 3A, 3O, 5C, 5G, 8E, 8P, and 5X (L-G2 and L-G3)]. Among these, isolates representing pathotype 5C appeared to be the most virulent on the rutabaga accessions, since only 0.8% (one of the 124 accessions) was resistant. In contrast, greater percentages (33.6, 62.4, and 66.4%) of the rutabaga accessions were resistant or moderately resistant to isolates representing the remaining three pathotypes (8J, 5L, and 5K, respectively) (Figure 2). There was no significant difference in the percentages of the rutabaga accessions R (5.6–8.8%), MR (5.6–9.6%), and S (84.0–84.8%) to the L-G2 and L-G3 isolates representing pathotype 5X.

**FIGURE 3:**
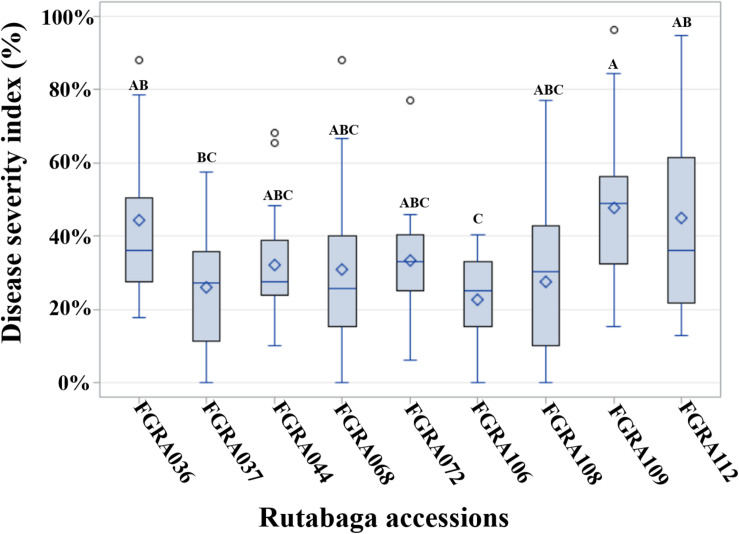
Distribution of indices of disease (IDs) among rutabaga (*Brassica napus* ssp. *napobrassica*) accessions resistant (R) or moderately resistant (MR) to 17 *Plasmodiophora brassicae* isolates representing 16 different pathotypes. The grand mean (GM) ID (♢), median (line inside box), 75th percentile (upper end of box), 25th percentile (lower end of box) as well as the maximum and minimum observations for all 17 isolates are presented by Box-and-Whiskers plots. The GM is the mean ID for an accession across all 17 isolates. The genotypes were considered R if the GM ID + Standard Deviation (SD) ≤ 30% and MR 30% < GM ID + SD ≤ 50%. Accessions with the same letters are not significantly different.

Resistance and moderate resistance were consistent among nine accessions (Figure 3 and Supplementary Table S3). Accession FGRA106 (GM ID = 22.7%) was resistant or moderately resistant to isolates representing all pathotypes (i.e., R to 11 and MR to 6 isolates). Three accessions FGRA037 (GM ID = 26.1%), FGRA108 (GM ID = 27.6%), and FGRA072 (GM ID = 33.3%) were resistant or moderately resistant to isolates representing 15 of the 16 pathotypes (i.e., R + MR to 16 of the 17 isolates) but were each susceptible to one pathotype; i.e., 3O, 8N and 8N, respectively. Two accessions, FGRA068 (GM ID = 31.0%) and FGRA044 (GM ID = 32.1%) were resistant to isolates representing 14 of the 16 pathotypes (i.e., R + MR to 15 of the 17 isolates) but susceptible to two pathotypes; 6M and 5C and 2F and 5X (L-G2), respectively. Another three accessions FGRA036 (GM ID = 44.5%), FGRA112 (GM ID = 45.0%), and FGRA109 (GM ID = 47.7%) exhibited considerable resistance or moderate resistance (i.e., R + MR to 9–11 of 17 isolates).

Overall, 12.1% of the rutabaga accessions were resistant or moderately resistant to ≥8 of the isolates, while the vast majority (87.9%) exhibited very little resistance (Figures 1–3). The latter comprised 33 (26.6%) accessions susceptible to all 17 *P. brassicae* isolates, and 76 (61.3%) accessions that were resistant or moderately resistant to isolates representing 1–7 pathotypes (data not shown). Therefore, the rutabaga accessions showed a wide variation in their reaction to isolates representing all the *P. brassicae* pathotypes used in this study. Resistance in the accessions with broad spectrum resistance was of the order FGRA106 > FGRA037 > FGRA108, FGRA068, FGRA044, FGRA072 > FGRA036, FGRA112 > FGRA109.

### Linkage Disequilibrium

**FIGURE 4:**
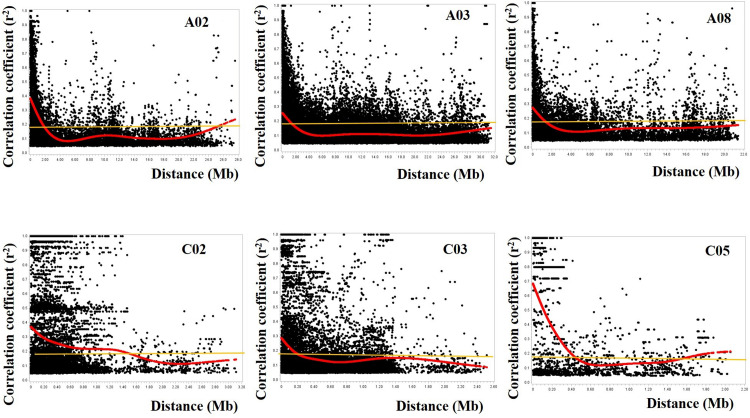
Plots of correlation coefficient (r^2^) as a function of physical distance (in Mb) between pairs of SNP markers on chromosomes A02, A03, and A08 of the A-genome (top) and chromosomes C02, C03, and C05 of the C-genome (bottom). Red curves represent the fit plots of the data points and the orange line represents the background linkage disequilibrium (BLD) or threshold line. The extent of LD decay was determined from projection of the intersection of the curves and the BLD line onto the physical distance. The SNP markers on the A-genome had a wider coverage (∼22–32 Mb) while a reduced set of SNP markers was available on the C-genome (∼2.0–3.2 Mb).

The deletion of the most distant SNP markers reduced the coverage length of chromosomes A04, C01, C02, C03, C04, C05, C06, C07, C08, and C09. For example, the deletion of just one or two SNPs from chromosomes C05, C06, C07, and C08 significantly reduced the SNP length covered from 19.9, 17.4, 26.6, and 33.0 Mb (data not shown) to 2.1, 3.2, 4.9, and 4.4 Mb, respectively (Table 1, Figure 4, and Supplementary Figures S1, S2). As a result, the 6751 SNP markers used for the estimation of LD decay covered 285.9 Mb (A-genome 259.1 Mb + C-genome 26.7 Mb) instead of 478.0 Mb (A-genome 265.9 Mb + C-genome 212.1 Mb, data not shown) if the 110 more distant SNP markers were included in the analysis. The marker density ranged from one marker/5.4–106.4 kb for the 19 chromosomes. The mean inter-marker distance were 63.4 ± 21.9 kb and 15.0 ± 8.4 kb for the A- and C-genomes, respectively. Overall, this translated to one SNP marker per 40.5 ± 29.8 kb in the rutabaga chromosomes (Table 1).

The average of the squared allele correlation LD (r^2^) for the A-genome, C-genome and the entire genome were calculated to be 0.041, 0.063, and 0.047, respectively (Table 1). About 22.3 ± 4.4% (A-genome 20.3 ± 3.4% + C-genome 24.5 ± 4.4%) of the total intra-chromosomal SNP pairs were in the significant LD (*p* < 0.001). Based on the selected significant SNPs, the background LD (estimated as the 95th percentile of unlinked *r*^2^-values) for the entire genome was determined to be r^2^ = 0.181. The average extent of LD decay (which was derived from the intersection between the fitted curve and background LD) for the 19 chromosomes ranged from 200 to 2300 kb (Figure 4 and Supplementary Figures S1, S2) with a mean of 1081.6 ± 649.9 kb (Table 1). The respective ranges for the A-and C-genome chromosomes were 1100–2300 kb and 200–1500 kb while the means were 1560.0 ± 408.8 kb and 550.0 ± 398.1 kb, respectively (Table 1). Based on the above data, LD in the C-genome decayed faster whereas LD persisted two to five times longer in the A-genome.

### Association Mapping of Clubroot Resistance Loci

**FIGURE 5:**
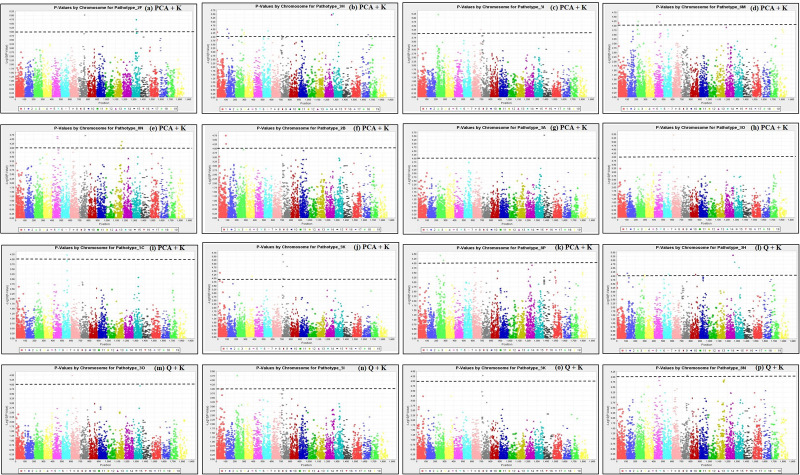
Manhattan plots of the PCA + K **(a–k)** and Q + K **(l–p)** models for identifying clubroot resistance loci in 124 rutabaga (*Brassica napus* ssp. *napobrassica*) accessions. The dashed horizontal lines indicate the Bonferroni-adjusted significance threshold (*P* ≤ 1 × 10^–4^). The dots above the significance threshold indicate SNPs associated with resistance to each *P*. *brassicae* isolate.

Analyses of the SNP marker genotype data with TASSEL 5.0 (Bradbury et al., 2007) detected population structure (Q) and kinship (K) among the 124 rutabaga accessions. Based on the Q-Q plots of the four GLM models, the deviation of the observed -log_10_ *P* distribution from the expected distribution was of the order Naïve > K-only > PCA-only and Q-only for both the PCR-based (data not shown) and SNP markers (Supplementary Figures S3A–D). All four MLM (Q + K, PCA + K, Q + K, and PCA + D) models (Supplementary Figures S3E–H) gave a minimal deviance of the observed -log_10_ *P* from the expected distribution compared with the aforementioned GLM models. In addition, the MLM models that utilized Kinship matrix (i.e., PCA + K and Q + K) departed the least from the expected distribution compared with the MLM models that utilized the Distance matrix (i.e., PCA + D and Q + D). Therefore, based on the Q-Q plots of the eight GWAS models, the PCA + K and Q + K MLM models were used for SNP-clubroot association analyses. Manhattan plots for the PCA + K and Q + K models used for the identification of clubroot resistance loci to the different isolates are presented in Figure 5.

Forty three (43) SNP markers were detected by the PCA + K model to be significantly associated with resistance to 11 *P. brassicae* pathotypes. This comprised 4, 13, 3, 4, 6, 2, 1, 3, 1, 5, and 1 SNPs which were associated with resistance to pathotypes 2F, 3H, 5I, 6M, 8N, 2B, 3A, 3O, 5C, 5K, and 8P, respectively (Table 2). The SNP marker Bn_A01_p161237 was significantly associated with resistance to pathotypes 3H and 6M, while the remaining 42 were associated with resistance to only one pathotype each. Thirteen (13) SNP markers were found by the Q + K model to be significantly associated with resistance to six *P. brassicae* pathotypes (Table 2). This comprised nine SNPs which were associated with resistance to pathotype 3H and one SNP marker each which was associated with resistance to pathotypes 3O, 5I, 5K, and 8N. Eleven of the 13 SNPs were detected both by the Q + K and PCA + K models, while two SNPs (Bn_A03_p21487106 and Bn_A05_p3191390) were detected only by the Q + K model (Table 2).

**TABLE 2:** SNP and PCR-based markers in rutabaga accessions, their chromosomal location and linkage association with clubroot caused by 17 single-spore and field isolates representing 16 different pathotypes of *Plasmodiophora brassicae*.

Model^θ^ Used	SNP Marker^α^	Marker physical position		Linkage group^β^	Pathotype*^Ω^*	Description of gene functions in *Brassica rapa, B. oleracea, B. napus*, and *Arabidopsis thaliana*^¥^
		
		Start	End			
PCA + K	Bn_A01_p161237	161177	161236	A01	3H and 6M	Molecular function unknwon
PCA + K	Bn_A01_p3070657^a^	3070658	3070717	A01	5K	Topoisomerase family protein
PCA + K	Bn_scaff_16394_1_p847636	23261545	23261593	A01	2F	Molecular function unknwon
PCA/Q + K	Bn_scaff_16876_1_p908602^b^	402290	402348	A02	3H	Nodulin-related; WAT1-related protein At5g40230-like
PCA/Q + K	Bn_A02_p4210188	4209928	4209987	A02	3H	Molecular function unknwon
PCA + K	Bn_A02_p6615821^c^	6615522	6615581	A02	3H	Molecular function unknwon
PCA/Q + K	Bn_A03_p21205471^d^	19989670	19989721	A02	3H	GDSL-motif lipase/hydrolase family protein
PCA + K	Bn_scaff_17721_1_p272248	25189709	25189756	A02	8N	Molecular function unknwon
PCA + K	Bn_A03_p7088375	7088115	7088174	A03	6M	EPIDERMAL patterning factor-like protein 6
PCA + K	Bn_A03_p7094698	7094346	7094430	A03	6M	Molecular function unknwon
PCA + K	Bn_A03_p8764481	8764218	8764282	A03	5I	Molecular function unknwon
PCA + K	Bn_A03_p13610858	13610459	13610518	A03	8P	WD-40 repeat family/beige-related; BEACH domain-containing protein
PCA + K	Bn_Scaffold000164_p55747^e^	16537330	16537377	A03	2B	Transcription activator; two-component response regulator-like APRR1
PCA + K	Bn_A07_p6850383^f^	19733579	19733672	A03	3O	Catalytic/cation binding/hydrolase
PCA/Q + K	Bn_A03_p21205471^d^	21204972	21205062	A03	3H	Histone deacetylase
PCA/Q + K	Bn_A03_p21377430	21376931	21377030	A03	3H	Molecular function unknwon
Q + K	Bn_A03_p21487106	21486607	21486666	A03	3H	Leucine-rich repeat receptor-like serine/threonine-protein kinase BAM2
PCA + K	Bn_scaff_16110_1_p2556157^g^	28182622	28182702	A03	3H	DDB1-CUL4 associated factor 1; nucleotide binding
PCA + K	Bn_A01_p3070657^a^	28201604	28201639	A03	5K	DNA topoisomerase family protein
PCA + K	Bn_A04_p248884	248621	248685	A04	3H	Molecular function unknwon
PCA + K	Bn_A04_p251383^h^	251033	251182	A04	3H	OTU-like cysteine protease family protein
PCA + K	Bn_A04_p15492182	15488727	15491658	A04	5K	Chaperone protein dnaJ 13; DNAJ heat shock N-terminal
PCA + K	Bn_scaff_16394_1_p842382	6023280	6023303	A04	2F	Condensin complex subunit 3
PCA + K	Bn_scaff_16394_1_p920749	6053461	6053519	A04	2F	Molecular function unknwon
PCA/Q + K	Bn_scaff_16876_1_p908602^b^	7580663	7580762	A04	3H	Transcriptional elongation regulator; WAT1-related protein At5g40230
PCA + K	Bn_scaff_15585_1_p978781^i^	15378727	15381200	A04	3O	PLL1 (POLTERGEIST LIKE 1); catalytic/protein serine/threonine phosphatase
PCA/Q + K	Bn_A05_p894768	894769	894868	A05	3H	DEAD/DEAH box helicase, putative; P-loop containing hydrolases protein
Q + K	Bn_A05_p3191390	3191391	3191450	A05	3H	Jacalin-related lectin 22-like
PCA + K	Bn_scaff_15585_1_p978781^i^	4971159	4973632	A05	3O	PLL1 (POLTERGEIST LIKE 1); catalytic/protein serine/threonine phosphatase
PCA + K	Bn_A05_p16738871	16737511	16737570	A05	6M	Translocation protein-related; nuclear transcription factor Y subunit A-9
PCA + K	Bn_A05_p17894045^j^	17892395	17892544	A05	6M	4-galactosyl-N-acetylglucosaminide 3-alpha-L-fucosyltransferase
PCA + K	Bn_A05_p19650278	19648618	19648677	A05	8N	Molecular function unknwon
PCA + K	Bn_A05_p19650965	19649366	19649425	A05	8N	Molecular function unknwon
PCA + K	Bn_A05_p17894045^j^	1871714	1871836	A06	6M	Fucosyltransferase, transferring glycosyl groups
PCA/Q + K	Bn_A06_p17037739	17036640	17036739	A06	5C	ATP binding/endoribonuclease, serine/threonine kinase
PCA/Q + K	Bn_A06_p18000461	17999362	17999421	A06	3H	Endonuclease, putative, Flap endonuclease
PCA + K	Bn_A07_p6850383^f^	6849583	6849682	A07	3O	BGLU9 (BETA GLUCOSIDASE 9); catalytic/cation binding/hydrolase
PCA + K	Bn_A07_p6542254^k^	6541555	6541704	A07	3O	Molecular function unknwon
PCA + K	Bn_A07_p22005058	22003808	22003957	A07	5I	Alpha-1,3/1,6-mannosyltransferase ALG2
PCA + K	Bn_A05_p17894045^j^	2515487	2515609	A08	6M	Fucosyltransferase, transferring glycosyl groups
PCA + K	Bn_A08_p8869180	8867982	8868079	A08	3H	SH3 domain-containing protein 1 (SH3P1)
PCA/Q + K	Bn_A08_p10123561	10122198	10122262	A08	5K	Molecular function unknwon
PCA + K	Bn_scaff_16110_1_p2556157^g^	13408834	13408889	A08	3H	DDB1-CUL4 Associated factor 1; nucleotide binding
PCA + K	Bn_A07_p6542254^k^	16843055	16843176	A08	3O	SYP61; syntaxin-61-like; SNAP receptor
PCA + K	Bn_A08_p17061248	17059485	17059549	A08	2F	Molecular function unknwon
PCA + K	Bn_A08_p17393018	17391255	17391319	A08	8N	Molecular function unknwon
PCA + K	Bn_A08_p18310412	18308549	18308613	A08	5K	Molecular function unknwon
PCA/Q + K	Bn_A03_p21205471^d^	2809099	2809170	A09	3H	Histone deacetylase
PCA + K	Bn_A07_p6542254^k^	11862168	11862292	A09	3O	SYP61 syntaxin-61-like; SNAP receptor
PCA + K	Bn_A04_p251383^h^	30512170	30512309	A09	3H	OTU-like cysteine protease family protein
PCA + K	Bn_A02_p6615821^c^	8176867	8176897	A10	3H	Molecular function unknwon
PCA/Q + K	Bn_scaff_16665_1_p188604	52668601	52668672	C03	3H	Molecular function unknwon
PCA/Q + K	Bn_scaff_16665_1_p199303	52679244	52679315	C03	3H	Molecular function unknown
PCA + K	Bn_scaff_17721_1_p273764	49814773	49814837	C02	8N	Vegetative storage protein 2
PCA + K	Bn_scaff_17721_1_p309137	49779344	49779408	C02	8N	Molecular function unknown
PCA/Q + K	Bn_scaff_18338_1_p871455	14173597	14173745	C05	2F, 5I and 3A	Molecular function unknown
PCA/Q + K	Bn_scaff_16876_1_p908602 ^*b*^	5278	5377	Scaffold000522	3H	Molecular function unknwon
PCA + K	Bn_Scaffold000247_p28610	28505	28613	Scaffold000247	5K	Molecular function unknwon
PCA + K	Bn_Scaffold000164_p46170	46171	46230	Scaffold000164	2B	ATMRP8; ATPase, coupled to transmembrane movement, ABC transporter C family member 6-like
PCA + K	Bn_Scaffold000164_p55747^e^	55748	55847	Scaffold000164	2B	Transcription activator; two-component response regulator-like APRR1
PCA + K	KB29N19 (SSR)	24637310	24637562	A03	3H, 6M, 8N, 2B, 3A, 5C, 5G and 5I	Molecular function unknown (Overlapping gene Bra019372)
PCA + K	B0903 (SSR)	24388788	24389001	A03	5C, 5L and 5K	Molecular function unknown (overlapping gene Bra019408)
PCA + K	B1005 (InDel)	24376817	24377055	A03	5K	Disease resistance protein (TIR-NBS-LRR class) (overlapping gene Bra019410)
PCA + K	A08-5021 (SSR)	11614477	11614809	A08	5L	Cyclase-associated protein 1-like (overlapping gene Bra034629)

*^θ^Mixed Linear Model (MLM) designations: PCA, principal component analysis; Q, population structure; K, Kinship. ^α^SNP markers denoted with the same superscript letter mapped to multiple chromosomes on the reference genomes. The type of PCR-based markers showing trait association has been specified. ^β^Linkage groups *A*_1_-*A*_10_ = Brassica rapa and *C*_1_-*C*_9_ = Brassica oleracea. ^Ω^Pathotype designations are as per the Canadian Clubroot Differential Set (letters) and the system of Williams (numbers). ^¥^Putative functions are based on matching entries in the GenBank database of NCBI.*

Only the PCA + K model was used in the case of the GWAS with the PCR-based markers. Two SSR markers KB29N19 and B0903 (Kato et al., 2012) on the *B. rapa* chromosome A03 were significantly associated with resistance to eight (3H, 6M, 8N, 2B, 3A, 5C, 5G, and 5I) and 3 pathotypes (5C, 5L, and 5K), respectively. The InDel marker B1005 (Kato et al., 2012), also on the A03 chromosome, was associated with resistance to pathotype 5K. In addition, the SSR marker A08-5021 (Hobson and Rahman, 2016) on the A08 chromosome was significantly associated with resistance to pathotype 5L. Therefore, a total of 45 (32 by PCA + K only, 2 by Q + K only and 11 by both PCA + K and Q + K) SNP markers and 4 PCR-based markers were detected to be significantly associated with resistance loci to the five old and eight of the 12 new *P. brassicae* pathotypes used in this study. None of the molecular markers were found to be associated with resistance to pathotypes 5X (L-G2 and L-G3), 8E or 8J.

**FIGURE 6:**
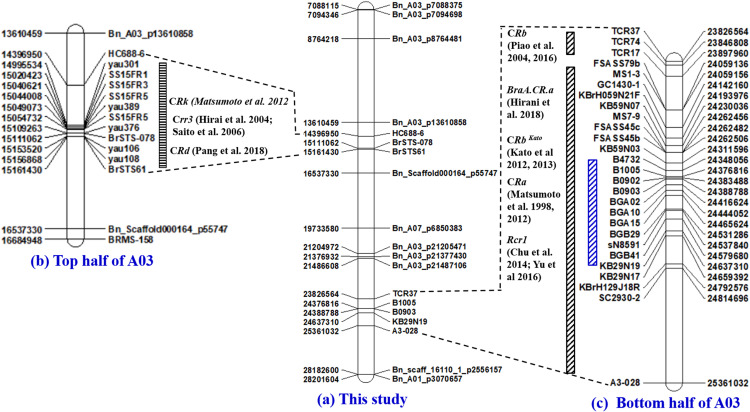
Physical maps of the A03 chromosome of *B. rapa* constructed by the use of SSR and SNP markers identified to be associated with clubroot resistance in this study **(a)** and the PCR-based markers previously identified to be linked to the *Crr3* (Hirai et al., 2004; Saito et al., 2006), *CRk* (Matsumoto et al., 2012), and *CRd* (Pang et al., 2018) gene(s) located on the top half of chromosome A03 **(b)**, as well as the *CRa* (Matsumoto et al., 1998, 2012), *CRb* (Piao et al., 2004; Zhang et al., 2014), *CRb*^Kato^ (Kato et al., 2012, 2013), *Rcr1* (Chu et al., 2014; Yu et al., 2016) and *BraA.CR.a* (Hirani et al., 2018) gene(s) located on the bottom half of chromosome A03 **(c)**.

**FIGURE 7:**
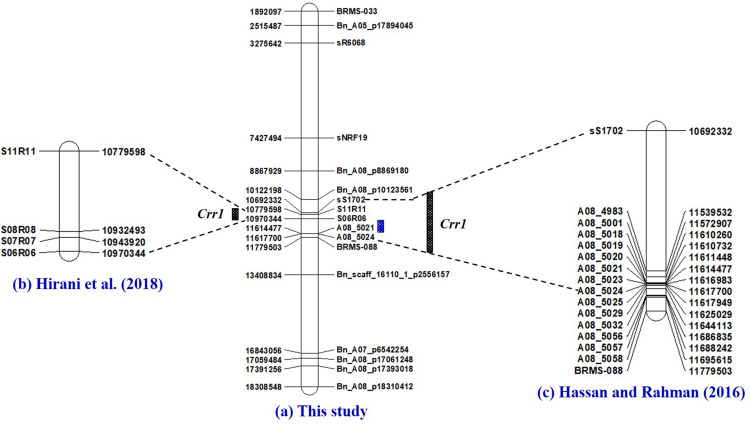
Physical maps of chromosome A08 of *Brassica rapa* constructed by the use of SSR and SNP markers identified to be associated with clubroot resistance in this study **(a)**, and the PCR-based markers previously identified to be linked to the *Crr1* gene (Hirani et al., 2018) **(b)** and Hasan and Rahman (2016) **(c)**. Fine mapping of the *Crr1* genomic region was conducted with SSR markers developed by Hobson and Rahman (2016).

About 85% of the markers identified mapped to the A-genome of *B. rapa* and *B. napus*. The highest number of markers (22%) mapped to the A03 chromosome, where the *Crr3* (Hirai et al., 2004; Saito et al., 2006), *CRk* (Matsumoto et al., 2012), *CRd* (Pang et al., 2018), *CRa* (Matsumoto et al., 1998, 2012), *CRb* (Piao et al., 2004; Zhang et al., 2014), *CRb*^Kato^ (Kato et al., 2012, 2013), *Rcr1* (Chu et al., 2014; Yu et al., 2016) and *BraA.CR.a* (Hirani et al., 2018) genes have been characterized previously (Figure 6). This next highest number of markers mapped to the A08 chromosome (13%), where the *Crr1* gene has been reported (Hatakeyama et al., 2013; Hasan and Rahman, 2016; Hirani et al., 2018; Figure 7). About 5–8% of the markers were identified on the A01, A02, and A06 chromosomes, where the *Crr2*, *CRc* and the *Crr4* genes have been identified, respectively. In addition, 11% of the markers mapped to each of the A04 and A05 chromosomes, while about 2–5% of the markers mapped to the A07, A09, and the A10 chromosomes, where no major clubroot resistance genes have been mapped thus far. About 8% of the markers mapped to the C-genome (C02, C03, and C05) of *B. oleracea*, while 7% mapped to scaffolds.

The three significant PCR-based markers (B1005, B0903, and KB2919) detected on the A03 chromosome were in LD with the closest SNP marker BnA03_P21487106 with *r*^2^-values in the range of 0.62–0.76. In the case of the significant SSR marker A08-5021 on the A08 chromosome, the *r*^2^-value with the closest SNP marker Bn_A08_p10123561 was 0.92. Thus, the four PCR-based markers and their closest SNP markers on both chromosomes were located on genomic regions tightly linked to CR genes. The physical positions and the chromosomal locations of the associated SNP and the PCR-based markers on the *B. rapa* and *B. oleracea* genome sequences are provided in Table 2.

### Putative Functions of Proteins Encoded by Identified Sequences

The sequences identified in this study matched entries in GenBank corresponding to ATP binding proteins, kinases, hydrolases, transferases, transcription factors, DNA topoisomerases, chaperone proteins, and translocation-related proteins, which are involved in cellular and biological processes as well as plant growth and development. Genes that encoded nucleotide-binding site leucine-rich repeat (NBS-LRR) proteins, WD-40 repeat family proteins, and syntaxin and histone deacetylases, which are associated with plant defense against pathogens, also matched some of the sequences identified in this study. Other matching genes encoded proteins including the RING/U-box superfamily proteins and nodulin-like transporter family proteins, which have been reported to play a role in plant growth and organ size development. Overall, about 60% of the genomic regions associated with the identified clubroot loci were previously characterized. The remaining 40% encoded proteins of unknown molecular function (Table 2).

## Discussion

The complex genetic basis of clubroot disease and the emergence of new virulent isolates of *P. brassicae* make it imperative to identify molecular markers significantly associated with resistance to different *P. brassicae* pathotypes for use in marker-assisted selection (MAS) and for the breeding of clubroot-resistant Brassica crops. Genome wide association studies have without doubt proven to be one of the most useful methods for finding significant marker-trait associations (Rafalski, 2010). In this study, 124 rutabaga accessions collected from Norway, Sweden, Finland, Denmark and Iceland were evaluated for resistance to pathotypes of 17 *P. brassicae* identified in Canada from 2003 to 2018 (Strelkov et al., 2005, 2016, 2018). This is the most comprehensive clubroot GWAS or genetic mapping study carried out to date, since the previous studies utilized from one to five pathotypes (Matsumoto et al., 1998, 2012; Hirai et al., 2004; Piao et al., 2004; Saito et al., 2006; Kato et al., 2012, 2013; Chu et al., 2014; Zhang et al., 2014; Fredua-Agyeman and Rahman, 2016; Hasan and Rahman, 2016; Li et al., 2016; Yu et al., 2016; Pang et al., 2018). Various studies have reported that transformation of data do not necessarily lead to the estimation of the correct Type 1 error rates (Beasley et al., 2009; Goh and Yap, 2009). Therefore, we used the untransformed data bearing in mind that Type 1 errors may be inflated. Similar, data analysis on the untransformed data was carried out by Zhou et al. (2018). However, comparing the power of different methods and determining the merits of whether or not to transform data to achieve normality may be beyond the scope of this study.

Linkage disequilibrium is important for determining the number and density of markers needed for GWAS and the experimental design needed to perform association analysis (Flint-Garcia and Thornsberry, 2003). The filtered set of 6751 SNP markers obtained from the *Brassica* 13.2K SNP array covered 285.9 Mb of the *B. napus* genome. About twice the coverage (∼645 Mb) has been reported in studies that have used the *Brassica* 60K array (Qian et al., 2014; Qu et al., 2017), as well as by the study by Zhou et al. (2017) that utilized Specific-Locus Amplified Fragment Sequencing (SLAF) technology. Despite using a reduced SNP array set (especially on the C-genome), the mean marker density (Table 1) of 14 of the 19 chromosomes in this study was within the range of one SNP/14.5–72.0 kb obtained by Qian et al. (2014), one SNP/25.0–52.9 kb according to Li et al. (2014) and one SNP/11.6–54.7 kb obtained by Qu et al. (2017) during association studies of *B. napus* using the *Brassica* 60K SNP array. Like the aforementioned studies, the marker density varied greatly among chromosomes and also according to genome.

Furthermore, the extent of LD decay using high density SNP markers in *B. napus* was reported by Qian et al. (2014) to vary from 80 to 2000 kb for the A-genome and from 400 to 7500 kb for the C-genome. Wu et al. (2016) reported the LD levels in the A-genome varied from 106 to 1908 kb whereas that in the C-genome varied from 227 to 4089 kb. However, Qu et al. (2017) and Zhou et al. (2017) reported much smaller ranges of LD decay for the A-genome (50–100 kb and 9–141 kb) and C-genome (1250–1500 kb and 63–1993 kb). Therefore, the determined LD decay in this study (which varied from 1100 to 2300 kb for the A-genome and which ranged from 200 to 1500 kb for C-genomes) was comparable to previous decayed LD estimates obtained in the aforementioned studies.

In *B. napus*, one cM was estimated to correspond to 500 kb (Suwabe et al., 2006; Ecke et al., 2010). Therefore, the total length of 285.9 Mb covered by the 6751 SNP markers correspond to approximately a quarter (571.7 cM) of the length of the genetic map of *B. napus* (∼2500 cM; Delourme et al., 2013). With the minimum LD decay of 200 kb (∼0.4 cM), a maximum of 1450 (few thousands) evenly spaced SNP markers would be necessary to perform the GWAS in the rutabaga accessions. Therefore, the more than 6000 SNP markers used for the GWAS should have sufficient power to perform a good association analysis. The variations in the LD decay among the 19 chromosomes were large and hence significant SNP-trait associations were extended to flanking regions 0.2 cM∼100 kb upstream and downstream of the identified genomic hotspots or candidate genes. Different GWAS models were tested to find the best fit model for identifying associations between the SNP and PCR-based markers and clubroot resistance. Clearly, the PCA + K model, which found 43 SNP markers to be strongly associated with clubroot resistance, overestimated the number of markers. In contrast the Q + K model, which detected 13 markers, underestimated the number of markers associated with clubroot resistance. Therefore, we combined the results from the two models to harness the strengths of the two methods. The 11 markers captured by the two MLM models were the most consistent, but we found that markers detected by either the PCA + K or the Q + K method only still had value. For example, the SNP marker Bn_A03_p21487106, which was detected only by the Q + K model, was associated with Leucine-rich repeat receptor kinases (LRR-RKs). The InDel marker B1005, which was detected only by the PCA + K model, was associated with the Toll and interleukin-1 receptor nucleotide binding site-Leucine rich repeat (TIR-NBS-LRR) protein. These disease resistance proteins play roles in host-specific and non-host-specific defense and wounding responses, as well as in the control of development, the stress response and senescence in plants (Torii, 2004; McHale et al., 2006; Passricha et al., 2018).

‘Electronic PCR’ (e-PCR) is a useful procedure to search for and position DNA sequences on reference genomes (Schuler, 1998; Rotmistrovsky et al., 2004; Fredua-Agyeman and Rahman, 2016). By positioning the markers identified in this study and markers from previous studies that co-segregated with clubroot resistance on the *B. rapa* reference genome sequences, we identified the top and bottom segments of the A03 chromosome and the middle segment of the A08 chromosome of rutabaga as genomic hotspots associated with resistance to the different *P. brassicae* pathotypes.

The first genomic hotspot was located at the top half of the A03 chromosome and was found by e-PCR to be flanked by the SCAR marker HC688-6 (14,396,950 nt) (Matsumoto et al., 2012) and the STS marker BrSTS-061 (15,161,430 nt) (Pang et al., 2018). In this study, the SNP markers Bn_A03_p13610858 (13,610,459 nt) and Bn_Scaffold000164 _p55747 (16,537,330 nt) flanked the *Crr3* (Hirai et al., 2004; Saito et al., 2006), *CRk* (Matsumoto et al., 2012), and *CRd* (Pang et al., 2018) genes. The SNP marker Bn_A03_p13610858 was located ∼786,000 nt upstream of the SCAR marker HC688-6, while the SNP marker Bn_Scaffold000164_p55747 was located ∼1,375,000 nt downstream of BrSTS61. The genomic region based on the SNP markers spanned ∼3,000,000 nt compared with that based on the PCR-based markers which spanned ∼765,000 nt and conferred resistance to the field isolates representing pathotypes 2B and 8P. The large physical distances between the SNP and the PCR-based markers located at the top half of the A03 chromosome, and the fact that the genes controlling clubroot resistance in this region conferred resistance to multiple *P. brassicae* pathotypes, suggest that the genes controlling clubroot resistance in this region are independent.

The second genomic hotspot was located at the bottom half of the A03 chromosome and conferred resistance to isolates representing 10 pathotypes. Fredua-Agyeman and Rahman (2016) identified the major resistance gene(s) in this genomic region to be the *CRa* and or the *CRb*^Kato^ genes based on markers from Matsumoto et al. (1998, 2012) and Kato et al. (2012, 2013). In the literature, three other clubroot resistance genes *CRb* (Piao et al., 2004; Zhang et al., 2014), *Rcr1* (Chu et al., 2014; Yu et al., 2016), and *BraA.CR.a* (Hirani et al., 2018) were also mapped to the same genomic region. Marker TCR37 (23,826,564 nt) (Zhang et al., 2014) and SC2930-2 (24,814,696 nt) (Matsumoto et al., 1998, 2012) flanked all five major clubroot resistance genes (*CRa*, *CRb*, *CRb*^Kato^, *Rcr1*, and *BraA.CR.a*) on the bottom half of the A03 chromosome and this genomic region spanned about 1 million (∼988,000 nt) nucleotides. In this study, a much smaller genomic region (∼260,000 nt) with flanking markers B1005 (24,376,816 nt) and KB29N19 (24637310 nt) conferred resistance to four SSIs (pathotypes 3H, 5I, 6M, and 8N) and six field isolates (pathotypes 2B, 3A, 5C, 5G, 5L, and 5K). In contrast to the top half, this study and the previous studies mapped the *CRa*, *CRb*, *CRb*^Kato^, *Rcr1*, and *BraA.CR.a* genes located at the bottom half of the A03 chromosome to <1 million nucleotides from each other. This strongly suggests that the aforementioned reported genes could be alleles. It also is possible, however, that they could be part of a gene cluster with each conferring resistance to the different pathotypes as reported by Zhang et al. (2014) and Yu et al. (2016).

The third genomic hotspot was located at the middle segment of the A08 chromosome with SSR marker A08-5021 (11,614,477 nt) being closest to the overlapping gene (Bra034629) and the SNP markers Bn_A08_p10123561 (10,122,198 nt) and Bn_scaff_16110_1_p2556157 (13,408,834) as the flanking markers. This genomic region conferred resistance to pathotypes and 3H, 5L and 5K in this study. Hirani et al. (2018) used linkage analysis to position the *Crr1* gene between SSR markers S11R11 and S06R06, which by e-PCR was between 10,779598 and 10,970,334 nt. Hasan and Rahman (2016) reported that the *Crr1* gene in the rutabaga cultivar ‘Brookfield’ was located in the genomic region (10,692,602–11,617,968 nt) and it conferred resistance to pathotypes 2F, 3H, 5I, 6M, and 8N. Unfortunately, e-PCR could not position any of the *Crr1* markers used by Hatakeyama et al. (2013) on the *B. rapa* genome. However, markers for the amplification of the 2nd Intron and 3rd intron of the *Crr1* gene were positioned between S11R11 (8,529,231 nt) and S27R27 (6,871,891 nt) on the *B. napus* genome. This study positioned the *Crr1* gene in the same genomic region as was reported by the other three studies. Hatakeyama et al. (2013) reported two gene loci for the *Crr1* gene: *Crr1a* which encodes a TIR-NB-LRR class of R proteins and has major effects, and *Crr1b* with minor effects but necessary to confer resistance to some *P. brassicae* isolates. The overlapping genes identified in this genomic region encoded a cyclase-associated protein, a DDB1-CUL4 associated factor and proteins of unknown molecular function. Therefore, functional analysis studies need to be carried out to get a full understanding of the role of the overlapping genes in this genomic region.

## Conclusion

In conclusion, the rutabaga accessions identified in this study can be used as new donor resistant germplasm for the next generation of CR *Brassica napus* variety development. This is because markers linked to clubroot resistance in the rutabaga accessions mapped to genomic regions on the A03 and A08 chromosomes where almost all CR genes (*CRk*, *Crr3*, *CRd*, *CRa*, *CRb*^Kato^, *Rcr1*, *CRb*, and *Crr1*) on the A-genome are housed. In addition, the study identified novel clubroot resistance loci on the C-genome of rutabaga associated with resistance to different *P. brassicae* pathotypes from Alberta. Markers identified in this study need to be validated to find out whether they directly co-segregate with clubroot resistance or they are in linkage disequilibrium with a QTL that contributes to the resistance. The markers identified in this study will be valuable in MAS and the breeding of clubroot resistant cruciferous crops.

## Data Availability Statement

The datasets generated for this study can be found in the text, figures, tables and Supplementary Material of the article.

## Author Contributions

RF-A: grant application, collection of germplasm, data analysis, writing of manuscript, and supervision of graduate student. ZY: seeding of plant materials, inoculum preparation, greenhouse inoculation experiments, washing of roots, disease rating, and writing of basic draft of manuscript. S-FH and SS: grant application, supervision and provision of technical support for all pathotyping work carried in the greenhouse, and revision of the manuscript.

## Conflict of Interest

The authors declare that the research was conducted in the absence of any commercial or financial relationships that could be construed as a potential conflict of interest.

## Footnotes

1 www.ncbi.nlm.nih.gov

## Supplementary Material

FIGURE S1 Plots of correlation coefficient (r^2^) vs. physical distance (Mb) for chromosomes A02, A04, A05, A06, A07, A09, and A10.

FIGURE S2 Plots of correlation coefficient (r^2^) vs. physical distance (Mb) for chromosomes C01, C04, C06, C07, C08, and C09.

FIGURE S3 Quantile-Quantile comparison of eight GWAS models for identifying clubroot resistance loci in 124 rutabaga (*Brassica napus* ssp. *napobrassica*) accessions tested with 17 field and single-spore isolates of *Plasmodiophora brassicae* representing 16 different pathotypes. The four GLM tested comprised the Naïve(N) **(A)**, Kinship (K)-only **(B)**, Population structure (Q)-only **(C)**, and the Principal Coordinate Analysis (PCA)-only **(D)**. The four MLM tested comprised Q + D **(E)**, Q + K **(F)**, PCA + D **(G)**, PCA + K **(H)** models; where D is the Distance Matrix. The black line is the expected -log_10_ *P* distribution while colored lines are the observed -log_10_ *P* distribution for each of the 17 *P. brassicae* pathotypes.

TABLE S1 List of 60 PCR-based primers linked to previously identified clubroot resistance genes used to genotype 124 rutabaga accessions.

TABLE S2 Descriptive statistics of clubroot resistance variations in rutababa accessions collected from Norway, Sweden, Finland, Denmark and Iceland.

TABLE S3 Summary: Reactions of 124 rutabaga (*Brassica napus* ssp. *napobrassica*) accessions to 17 Plasmodiophora brassicae isolates.
