# Supplementary material for: Genome-Wide Mapping of Loci Associated With Resistance to Clubroot in Brassica napus ssp. napobrassica (Rutabaga) Accessions From Nordic Countries
Source: Front Plant Sci. 2020 Jun 12;11:742. doi: 10.3389/fpls.2020.00742 (PMC7303339; doi:10.3389/fpls.2020.00742)
Supplement: FIGURE S1 — Plots of correlation coefficient (r2) vs. physical distance (Mb) for chromosomes A02, A04, A05, A06, A07, A09, and A10. [file Table_2.docx]

**Table S2**. Descriptive statistics of clubroot resistance variations in rutababa accessions collected from Norway, Sweden, Finland, Denmark and Iceland.

| Pathotype | Mean ± sd | Mode | Min (%) | 50% Quantile (%) | Max (%) | Coefficient of variation (%) |
| --- | --- | --- | --- | --- | --- | --- |
| 2F | 78.7 ± 27.2 | 100.0 | 6.3 | 90.3 | 100.0 | 34.4 |
| 3H | 83.6 ± 27.1 | 100.0 | 0.0 | 100.0 | 100.0 | 32.3 |
| 5I | 82.9 ± 26.8 | 100.0 | 0.0 | 100.0 | 100.0 | 32.2 |
| 6M | 81.5 ± 27.2 | 100.0 | 1.6 | 95.8 | 100.0 | 33.2 |
| 8N | 82.9 ± 27.2 | 100.0 | 4.2 | 95.8 | 100.0 | 32.7 |
| 5X (LG2) | 73.6 ± 29.8 | 100.0 | 3.7 | 83.3 | 100.0 | 40.3 |
| 5X (LG3) | 63.8 ± 29.9 | 100.0 | 5.6 | 72.2 | 100.0 | 46.7 |
| 5L | 30.2 ± 34.0 | 100.0 | 0.0 | 14.3 | 100.0 | 112.1 |
| 3A | 77.5 ± 27.8 | 100.0 | 0.0 | 87.5 | 100.0 | 35.8 |
| 2B | 80.6 ± 28.2 | 100.0 | 0.0 | 97.9 | 100.0 | 34.8 |
| 5G | 78.9 ± 28.0 | 100.0 | 4.6 | 95.1 | 100.0 | 35.4 |
| 8E | 77.2 ± 30.4 | 100.0 | 0.0 | 97.9 | 100.0 | 39.2 |
| 5C | 82.4 ± 22.8 | 100.0 | 20.6 | 93.1 | 100.0 | 27.6 |
| 8J | 56.3 ± 38.6 | 100.0 | 0.0 | 63.1 | 100.0 | 68.4 |
| 5K | 30.3 ± 34.7 | 100.0 | 0.0 | 13.2 | 100.0 | 114.2 |
| 3O | 70.7 ± 32.1 | 100.0 | 5.6 | 87.5 | 100.0 | 45.2 |
| 8P | 69.7 ± 33.6 | 100.0 | 0.0 | 83.3 | 100.0 | 48.0 |

**Table S3.** Summary: Reactions of 124 rutabaga (*Brassica napus* ssp. *napobrassica*) accessions to 17 *Plasmodiophora brassicae* isolates.

| Accession | 2F | 3H | 5I | 6M | 8N | 5X^1^ | 5X^2^ | 5L | 2B | 3A | 5C | 5G | 8E | 5K | 8J | 3O | 8P | All Pb | R | MR | S | *Crr1* | *Crr3* | *CRb* | *CRa / CRb^Kato^ / Rcr1* |
| --- | --- | --- | --- | --- | --- | --- | --- | --- | --- | --- | --- | --- | --- | --- | --- | --- | --- | --- | --- | --- | --- | --- | --- | --- | --- |
| FGRA001 | S | S | S | S | S | S | S | S | S | S | S | S | S | S | S | S | S | S | 0 | 0 | 17 | - | - | - | - |
| FGRA002 | S | S | S | S | S | S | R | R | S | S | S | S | S | R | R | S | S | S | 4 | 0 | 13 | - | - | - | - |
| FGRA003 | MR | S | R | R | R | S | S | MR | S | R | S | S | MR | S | S | R | R | S | 6 | 3 | 8 | - | - | - | - |
| FGRA004 | S | S | S | S | S | S | S | MR | S | S | S | S | S | MR | S | S | S | S | 0 | 2 | 15 | - | - | - | - |
| FGRA005 | S | S | S | S | S | S | S | S | S | S | S | S | S | S | S | S | S | S | 0 | 0 | 17 | - | - | - | - |
| FGRA006 | S | S | S | S | S | S | S | MR | S | S | S | S | S | S | S | S | S | S | 0 | 1 | 16 | - | - | - | - |
| FGRA007 | S | S | S | S | S | S | S | MR | S | S | S | S | S | R | MR | S | S | S | 1 | 2 | 14 | - | - | - | - |
| FGRA008 | S | S | S | S | S | S | S | S | S | S | S | S | S | R | S | S | S | S | 1 | 0 | 16 | - | - | - | - |
| FGRA009 | S | S | S | S | S | S | S | S | S | S | S | S | S | S | S | S | S | S | 0 | 0 | 17 | - | - | - | - |
| FGRA010 | S | S | S | S | S | S | S | R | S | S | S | S | S | S | S | S | S | S | 1 | 0 | 16 | - | - | - | - |
| FGRA011 | S | S | S | MR | MR | MR | S | S | MR | S | S | S | MR | MR | S | MR | S | S | 0 | 7 | 10 | - | - | - | - |
| FGRA012 | S | S | S | S | S | S | S | R | S | S | S | S | S | R | MR | S | S | S | 2 | 1 | 14 | - | - | - | - |
| FGRA013 | S | S | S | S | S | S | S | S | S | S | S | S | S | S | S | S | S | S | 0 | 0 | 17 | - | - | - | - |
| FGRA014 | S | S | S | S | S | S | S | R | S | S | S | S | S | R | S | S | S | S | 2 | 0 | 15 | - | - | - | - |
| FGRA015 | S | S | S | S | S | S | S | S | S | S | S | S | S | R | S | S | S | S | 1 | 0 | 16 | - | - | - | - |
| FGRA016 | S | S | S | S | S | S | S | S | S | S | S | S | S | S | S | S | S | S | 0 | 0 | 17 | - | - | - | - |
| FGRA017 | S | S | S | S | S | S | S | S | S | S | S | S | S | MR | S | S | S | S | 0 | 1 | 16 | - | - | - | - |
| FGRA018 | S | S | S | S | R | R | MR | R | S | MR | S | MR | S | R | R | MR | R | S | 6 | 4 | 7 | - | - | - | - |
| FGRA019 | S | S | S | S | S | S | S | R | S | S | S | S | S | R | S | MR | S | S | 2 | 1 | 14 | - | - | - | - |
| FGRA020 | S | S | S | S | S | S | S | R | S | S | S | S | S | MR | S | S | S | S | 1 | 1 | 15 | - | - | - | - |
| FGRA021 | S | S | S | S | S | S | S | S | S | S | S | S | S | S | S | S | S | S | 0 | 0 | 17 | - | - | - | - |
| FGRA022 | S | S | S | S | S | S | S | S | S | S | S | S | S | S | S | S | S | S | 0 | 0 | 17 | - | - | - | - |
| FGRA023 | S | S | S | S | S | S | MR | R | S | S | S | S | S | R | R | MR | S | S | 3 | 2 | 12 | - | - | - | - |
| FGRA024 | S | S | S | S | S | S | S | S | S | S | S | S | S | S | S | S | S | S | 0 | 0 | 17 | - | - | - | - |
| FGRA025 | S | S | S | S | S | S | S | S | S | S | S | S | S | MR | S | S | S | S | 0 | 1 | 16 | - | - | - | - |
| FGRA026 | S | S | S | S | S | S | S | S | S | S | S | S | S | S | S | S | S | S | 0 | 0 | 17 | - | - | - | - |
| FGRA027 | S | S | S | S | S | S | S | R | MR | S | S | S | S | MR | R | S | S | S | 2 | 2 | 13 | - | - | - | - |
| FGRA028 | S | S | S | S | S | S | S | R | S | S | S | S | S | R | MR | S | S | S | 2 | 1 | 14 | - | - | - | - |
| FGRA029 | S | S | S | S | S | S | S | S | S | S | S | S | S | MR | S | S | S | S | 0 | 1 | 16 | - | - | - | - |
| FGRA030 | S | MR | MR | S | S | MR | S | R | S | S | S | MR | S | R | R | S | S | S | 3 | 4 | 10 | - | - | - | - |
| FGRA031 | S | S | S | S | S | S | S | S | S | S | S | S | S | S | S | S | S | S | 0 | 0 | 17 | - | - | - | - |
| FGRA032 | S | S | S | S | S | S | S | R | S | S | S | S | S | R | S | S | S | S | 2 | 0 | 15 | - | - | - | - |
| FGRA033 | S | S | S | S | S | S | S | R | S | S | S | S | S | R | R | S | S | S | 3 | 0 | 14 | - | - | - | - |
| FGRA034 | S | S | S | S | S | S | S | R | S | S | S | S | S | R | R | S | S | S | 3 | 0 | 14 | - | - | - | - |
| FGRA035 | S | S | S | S | S | S | S | MR | S | S | S | S | S | R | MR | S | S | S | 1 | 2 | 14 | - | - | - | - |
| FGRA036 | S | S | S | MR | MR | MR | S | R | MR | R | S | R | R | R | S | MR | MR | MR | 5 | 6 | 6 | + | - | - | + |
| FGRA037 | R | R | R | R | MR | R | MR | MR | MR | R | MR | R | MR | R | R | S | R | R | 10 | 6 | 1 | + | + | + | + |
| FGRA038 | S | S | S | S | S | S | S | S | S | S | S | S | S | MR | R | S | S | S | 1 | 1 | 15 | - | - | - | - |
| FGRA039 | MR | S | S | S | S | S | S | MR | S | S | S | S | S | MR | R | S | S | S | 1 | 3 | 13 | - | - | - | - |
| FGRA040 | S | S | S | MR | S | S | S | R | MR | S | S | S | S | MR | S | S | MR | S | 1 | 4 | 12 | - | - | - | - |
| FGRA041 | MR | MR | R | R | R | S | S | S | S | R | S | MR | MR | S | S | R | R | S | 6 | 4 | 7 | - | - | - | - |
| FGRA042 | S | S | S | S | S | S | S | R | S | S | S | S | S | R | R | S | S | S | 3 | 0 | 14 | - | - | - | - |
| FGRA043 | S | S | S | S | S | S | S | MR | S | S | S | S | S | S | S | S | S | S | 0 | 1 | 16 | - | - | - | - |
| FGRA044 | S | R | MR | MR | R | S | R | MR | MR | MR | R | MR | R | R | R | R | R | R | 9 | 6 | 2 | + | + | + | + |
| FGRA045 | S | S | S | S | S | S | S | R | S | S | S | S | S | MR | S | S | S | S | 1 | 1 | 15 | - | - | - | - |
| FGRA046 | S | S | S | S | S | S | S | S | S | S | S | S | S | S | S | S | S | S | 0 | 0 | 17 | - | - | - | - |
| FGRA047 | S | S | S | S | S | S | R | R | S | S | S | MR | S | S | S | MR | R | S | 3 | 2 | 12 | - | - | - | - |
| FGRA048 | S | S | S | S | S | S | S | S | S | S | S | S | S | S | S | S | S | S | 0 | 0 | 17 | - | - | - | - |
| FGRA049 | S | S | S | S | S | S | S | MR | S | S | S | S | S | S | S | S | S | S | 0 | 1 | 16 | - | - | - | - |
| FGRA050 | S | S | S | S | S | S | R | R | S | S | S | S | S | R | R | MR | S | S | 4 | 1 | 12 | - | - | - | - |
| FGRA051 | S | S | S | S | S | S | S | S | S | S | S | S | S | S | S | S | S | S | 0 | 0 | 17 | - | - | - | - |
| FGRA052 | S | S | S | S | S | S | S | MR | S | S | S | S | S | R | S | S | S | S | 1 | 1 | 15 | - | - | - | - |
| FGRA053 | S | S | S | S | S | S | S | R | S | S | S | S | S | R | R | S | S | S | 3 | 0 | 14 | - | - | - | - |
| FGRA054 | S | S | S | S | S | S | S | MR | S | S | S | S | S | R | MR | S | S | S | 1 | 2 | 14 | - | - | - | - |
| FGRA055 | S | S | S | S | S | S | S | S | S | S | S | S | S | S | S | S | S | S | 0 | 0 | 17 | - | - | - | - |
| FGRA056 | S | S | S | S | S | S | MR | MR | S | S | S | S | S | R | MR | S | S | S | 1 | 3 | 13 | - | - | - | - |
| FGRA057 | S | S | S | S | S | S | S | S | S | S | S | S | S | S | S | S | S | S | 0 | 0 | 17 | - | - | - | - |
| FGRA058 | MR | S | MR | S | S | R | MR | R | S | R | S | S | R | R | R | R | MR | S | 7 | 4 | 6 | - | - | - | - |
| FGRA059 | S | S | S | S | S | R | S | R | S | S | S | S | S | S | S | MR | MR | S | 2 | 2 | 13 | - | - | - | - |
| FGRA060 | S | S | S | S | S | S | S | R | S | S | S | S | S | R | S | S | S | S | 2 | 0 | 15 | - | - | - | - |
| FGRA061 | S | S | S | MR | S | MR | R | R | S | S | S | S | S | R | R | S | R | S | 5 | 2 | 10 | - | - | - | - |
| FGRA062 | S | S | S | S | S | S | S | S | S | S | S | S | S | R | S | S | S | S | 1 | 0 | 16 | - | - | - | - |
| FGRA063 | S | S | S | S | S | S | S | R | S | S | S | S | S | MR | S | S | S | S | 1 | 1 | 15 | - | - | - | - |
| FGRA064 | S | S | S | S | S | S | S | MR | S | S | S | S | S | MR | S | S | S | S | 0 | 2 | 15 | - | - | - | - |
| FGRA065 | S | S | S | S | S | S | S | R | S | S | S | S | S | MR | S | S | S | S | 1 | 1 | 15 | - | - | - | - |
| FGRA066 | S | S | S | S | S | S | S | MR | S | S | S | S | S | R | R | S | S | S | 2 | 1 | 14 | - | - | - | - |
| FGRA067 | S | S | S | S | S | S | S | R | S | S | S | S | S | R | S | S | S | S | 2 | 0 | 15 | - | - | - | - |
| FGRA068 | MR | MR | MR | S | MR | R | R | R | R | MR | S | R | R | R | R | MR | R | MR | 9 | 6 | 2 | + | - | - | + |
| FGRA069 | S | S | S | S | S | S | S | S | S | S | S | S | S | S | S | S | S | S | 0 | 0 | 17 | - | - | - | - |
| FGRA070 | R | MR | S | R | S | S | S | S | S | S | S | S | S | MR | R | S | S | S | 3 | 2 | 12 | - | - | - | - |
| FGRA071 | S | S | S | S | S | S | S | S | S | S | S | S | S | S | S | S | S | S | 0 | 0 | 17 | - | - | - | - |
| FGRA072 | R | R | MR | MR | S | R | R | MR | MR | MR | MR | MR | MR | R | MR | MR | R | R | 6 | 10 | 1 | + | - | - | + |
| FGRA073 | S | S | S | S | S | S | S | S | S | S | S | S | S | S | S | S | S | S | 0 | 0 | 17 | - | - | - | - |
| FGRA074 | S | S | S | S | S | S | S | S | S | S | S | S | S | S | S | S | S | S | 0 | 0 | 17 | - | - | - | - |
| FGRA075 | S | S | S | S | S | S | S | R | S | S | S | S | S | R | S | S | S | S | 2 | 0 | 15 | - | - | - | - |
| FGRA076 | S | S | S | S | S | S | S | R | S | S | S | S | S | R | MR | S | S | S | 2 | 1 | 14 | - | - | - | - |
| FGRA077 | S | S | S | S | S | S | S | S | S | S | S | S | S | S | S | S | S | S | 0 | 0 | 17 | - | - | - | - |
| FGRA078 | S | S | S | S | S | R | MR | R | S | MR | MR | S | S | R | R | MR | MR | S | 4 | 5 | 8 | - | - | - | - |
| FGRA079 | S | S | S | S | S | MR | S | R | S | S | S | S | S | R | R | S | R | S | 4 | 1 | 12 | - | - | - | - |
| FGRA080 | S | S | S | S | S | S | S | R | S | S | S | S | S | R | R | S | S | S | 3 | 0 | 14 | - | - | - | - |
| FGRA081 | S | S | S | S | S | S | S | S | S | S | S | S | S | S | S | S | S | S | 0 | 0 | 17 | - | - | - | - |
| FGRA082 | S | S | S | S | S | S | S | MR | S | S | S | S | S | R | S | S | S | S | 1 | 1 | 15 | - | - | - | - |
| FGRA083 | S | S | S | S | S | MR | S | R | MR | MR | S | MR | MR | R | S | MR | MR | S | 2 | 7 | 8 | - | - | - | - |
| FGRA084 | S | S | S | S | S | S | S | S | S | S | S | S | S | MR | S | S | S | S | 0 | 1 | 16 | - | - | - | - |
| FGRA085 | S | S | S | S | S | S | S | S | S | S | S | S | S | S | S | S | S | S | 0 | 0 | 17 | - | - | - | - |
| FGRA086 | S | S | S | S | S | S | S | S | S | S | S | S | S | S | S | S | S | S | 0 | 0 | 17 | - | - | - | - |
| FGRA087 | S | S | S | S | S | S | S | R | S | S | S | S | S | R | S | S | S | S | 2 | 0 | 15 | - | - | - | - |
| FGRA088 | S | S | S | S | S | S | S | R | S | S | S | S | S | R | R | S | S | S | 3 | 0 | 14 | - | - | - | - |
| FGRA089 | S | S | S | S | S | S | S | R | S | S | S | S | S | R | S | S | S | S | 2 | 0 | 15 | - | - | - | - |
| FGRA090 | S | S | S | S | S | S | S | R | S | S | S | S | S | MR | MR | S | S | S | 1 | 2 | 14 | - | - | - | - |
| FGRA091 | S | S | S | S | S | S | S | R | S | S | S | S | S | R | S | S | S | S | 2 | 0 | 15 | - | - | - | - |
| FGRA092 | S | S | S | S | S | S | S | S | S | S | S | S | S | S | S | S | S | S | 0 | 0 | 17 | - | - | - | - |
| FGRA093 | S | S | S | S | S | S | S | S | S | S | S | S | S | S | S | S | S | S | 0 | 0 | 17 | - | - | - | - |
| FGRA094 | S | S | S | S | S | S | S | S | S | S | S | S | S | S | S | S | S | S | 0 | 0 | 17 | - | - | - | - |
| FGRA095 | S | S | S | S | S | S | S | S | S | S | S | S | S | MR | S | S | S | S | 0 | 1 | 16 | - | - | - | - |
| FGRA096 | S | S | S | S | S | S | S | S | S | S | S | S | S | S | S | S | S | S | 0 | 0 | 17 | - | - | - | - |
| FGRA097 | S | S | S | S | S | S | S | R | S | S | S | S | S | R | S | S | S | S | 2 | 0 | 15 | - | - | - | - |
| FGRA098 | S | S | S | S | S | S | S | S | S | S | S | S | S | S | S | S | S | S | 0 | 0 | 17 | - | - | - | - |
| FGRA099 | S | S | S | S | S | S | S | R | S | S | S | S | S | R | S | S | S | S | 2 | 0 | 15 | - | - | - | - |
| FGRA100 | S | S | S | S | S | S | S | S | S | S | S | S | S | S | S | S | S | S | 0 | 0 | 17 | - | - | - | - |
| FGRA101 | S | S | S | S | S | S | S | S | S | S | S | S | S | S | S | S | S | S | 0 | 0 | 17 | - | - | - | - |
| FGRA102 | S | S | S | S | S | S | S | S | S | S | S | S | S | S | S | S | S | S | 0 | 0 | 17 | - | - | - | - |
| FGRA103 | S | S | S | S | S | S | R | R | S | S | S | S | MR | MR | R | S | S | S | 3 | 2 | 12 | - | - | - | - |
| FGRA104 | S | S | S | S | S | S | S | R | S | S | S | S | S | R | S | S | S | S | 2 | 0 | 15 | - | - | - | - |
| FGRA105 | S | S | S | S | S | S | S | MR | S | S | S | S | S | MR | S | S | S | S | 0 | 2 | 15 | - | - | - | - |
| FGRA106 | R | MR | R | R | MR | MR | R | R | R | R | MR | MR | R | R | MR | R | R | R | 11 | 6 | 0 | + | - | - | + |
| FGRA107 | S | S | S | S | S | S | S | MR | S | S | S | S | S | R | MR | S | S | S | 1 | 2 | 14 | - | - | - | - |
| FGRA108 | MR | R | R | MR | S | MR | R | MR | R | R | MR | MR | R | MR | R | R | MR | R | 8 | 8 | 1 | + | - | - | + |
| FGRA109 | MR | S | S | S | S | MR | S | R | MR | S | MR | S | MR | R | S | MR | R | MR | 3 | 6 | 8 | + | - | - | + |
| FGRA110 | R | MR | MR | S | S | MR | S | R | S | S | S | S | S | MR | MR | S | S | S | 2 | 5 | 10 | - | - | - | - |
| FGRA111 | MR | MR | S | MR | S | S | S | S | S | S | S | S | S | MR | S | MR | S | S | 0 | 5 | 12 | - | - | - | - |
| FGRA112 | MR | S | S | MR | R | S | S | R | MR | S | S | MR | R | R | S | R | R | MR | 6 | 4 | 7 | + | - | - | + |
| FGRA113 | S | S | S | S | S | S | S | R | S | S | S | S | S | R | R | MR | MR | S | 3 | 2 | 12 | - | - | - | - |
| FGRA114 | S | S | S | S | S | S | R | R | S | S | S | S | S | R | R | S | S | S | 4 | 0 | 13 | - | - | - | - |
| FGRA115 | S | S | S | S | S | S | MR | MR | S | S | S | S | S | R | S | S | S | S | 1 | 2 | 14 | - | - | - | - |
| FGRA116 | S | S | S | S | S | S | S | R | S | S | S | S | S | MR | S | S | S | S | 1 | 1 | 15 | - | - | - | - |
| FGRA117 | S | S | S | S | S | S | S | MR | S | S | S | S | S | R | S | S | S | S | 1 | 1 | 15 | - | - | - | - |
| FGRA118 | S | S | S | S | S | S | S | R | S | S | S | S | S | R | R | S | MR | S | 3 | 1 | 13 | - | - | - | - |
| FGRA119 | S | S | S | S | S | S | S | R | S | S | S | S | S | R | MR | S | S | S | 2 | 1 | 14 | - | - | - | - |
| FGRA120 | S | S | S | S | S | MR | S | R | S | S | S | S | S | R | R | S | S | S | 3 | 1 | 13 | - | - | - | - |
| FGRA121 | S | S | S | S | S | MR | S | R | MR | S | S | S | MR | R | S | S | S | S | 2 | 3 | 12 | - | - | - | - |
| FGRA122 | S | S | S | S | S | S | S | R | S | S | S | S | S | MR | S | S | S | S | 1 | 1 | 15 | - | - | - | - |
| FGRA123 | S | S | S | S | S | S | S | S | S | S | S | S | S | S | S | S | S | S | 0 | 0 | 17 | - | - | - | - |
| FGRA124 | S | S | S | S | S | S | S | S | S | S | S | S | S | S | S | S | S | S | 0 | 0 | 17 | - | - | - | - |

^α^ Pathotype designations are based on the systems of the Canadian Clubroot Differential (CCD) set (Strelkov et al. 2018). Pathotypes 2F, 3H, 5I, 6M and 8N are single-spore isolates identified prior to the introduction of clubroot resistant (CR) varieties in Canada, while pathotypes 5X (5X^1^ = LG2 and 5X^2^ = LG3), 5L, 3A, 2B, 5G, 8E, 5C, 8J, 5K, 3O and 8P are represented by field isolates identified after the introduction of CR varieties in Canada.

^β^ The genotypes were considered R if the disease severity index (ID) + Standard Deviation (SD) ≤ 30%, MR 30% < ID + SD ≤ 50% and S ID + SD > 50%.

“+” indicates detection of alleles linked to the *Crr1* (Suwabe et al. 2006; Hatakeyama et al. 2013; Hassan and Rahman 2016; Hobson and Rahman 2016; Hirani et al. 2018) gene on chromosome A08 of *Brassica rapa* and for the Crr3 (Hirai et al. 2004; Saito et al. 2006), *CRb* (Piao et al. 2004; Zhang et al. 2014), C*Ra* (Matsumoto et al. 1998, 2012), *CRb*^Kato^ (Kato et al. 2012, 2013), *Rcr1* (Chu et al. 2014; Yu et al. 2016) gene(s) located on the chromosome A03 using PCR-based markers from aforementioned studies.

“-” does not necessarily mean that absence of a major gene or QTLs associated with resistance in an accession but may be rather due to lack of polymorphism in the markers used in this study.


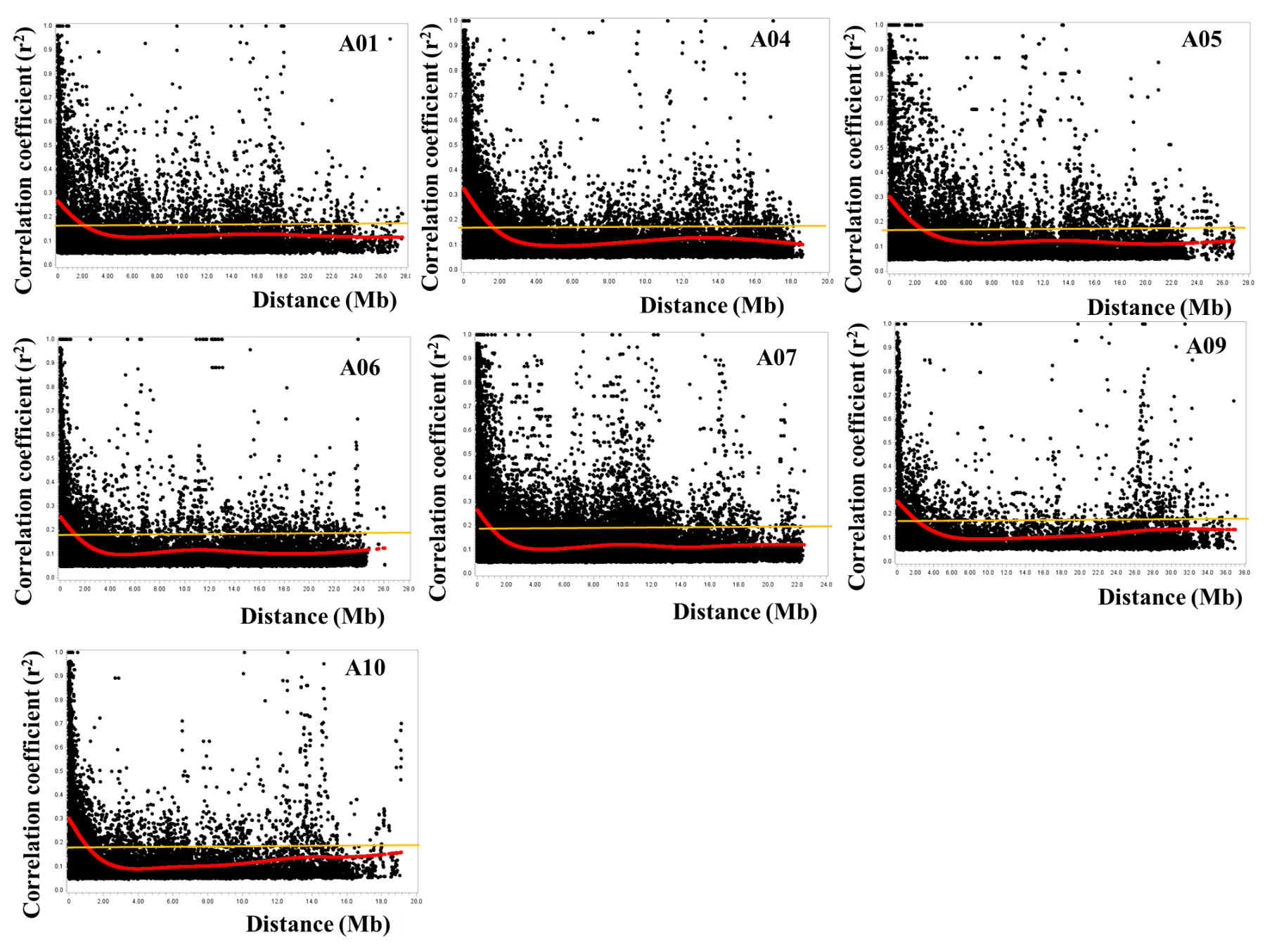


**Fig S1.** Plots of correlation coefficient (r^2^) vs. physical distance (Mb) for chromosomes A02, A04, A05, A06, A07, A09 and A10.


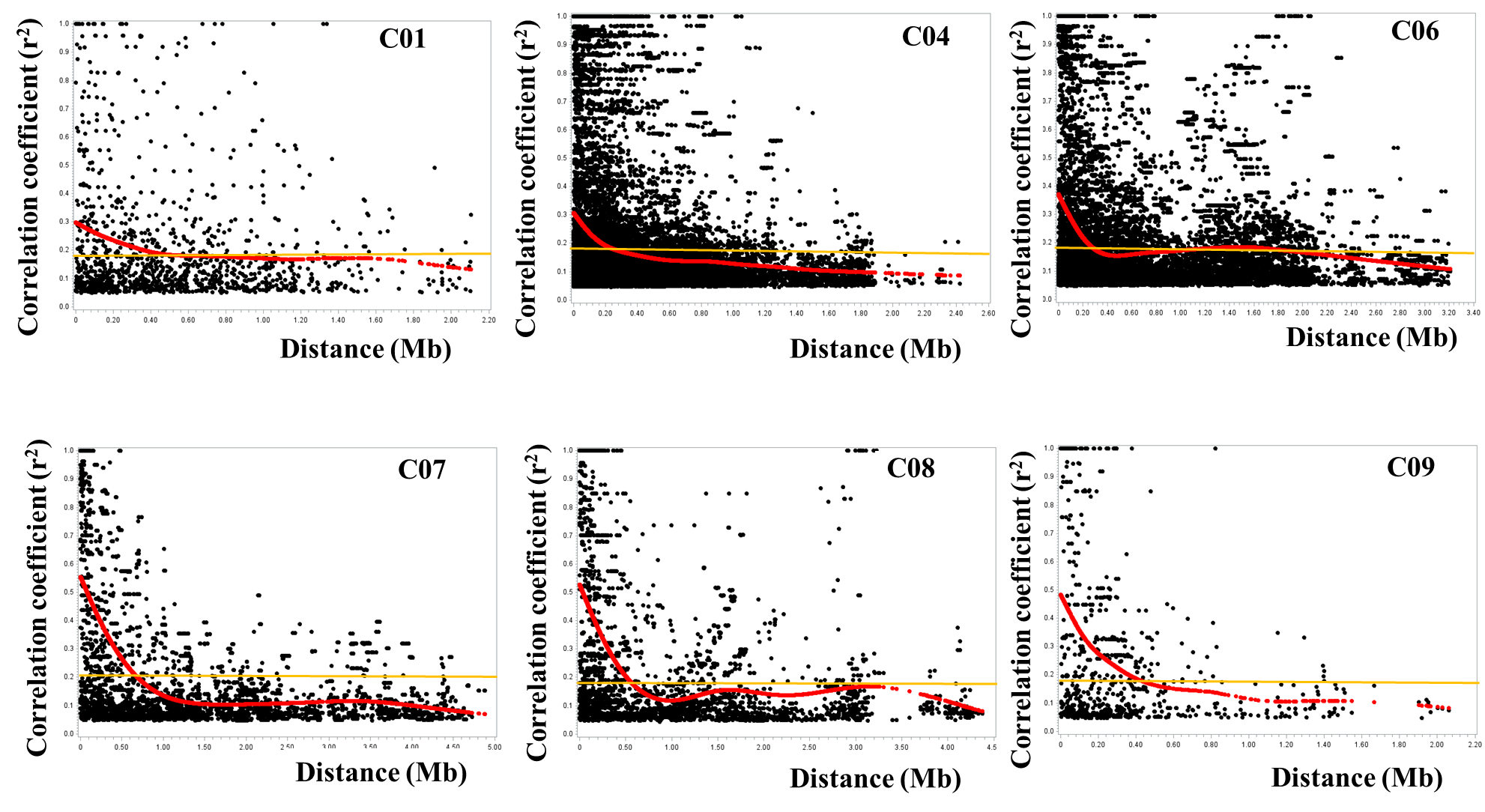


**Fig S2.** Plots of correlation coefficient (r^2^) vs. physical distance (Mb) for chromosomes C01, C04, C06, C07, C08 and C09.


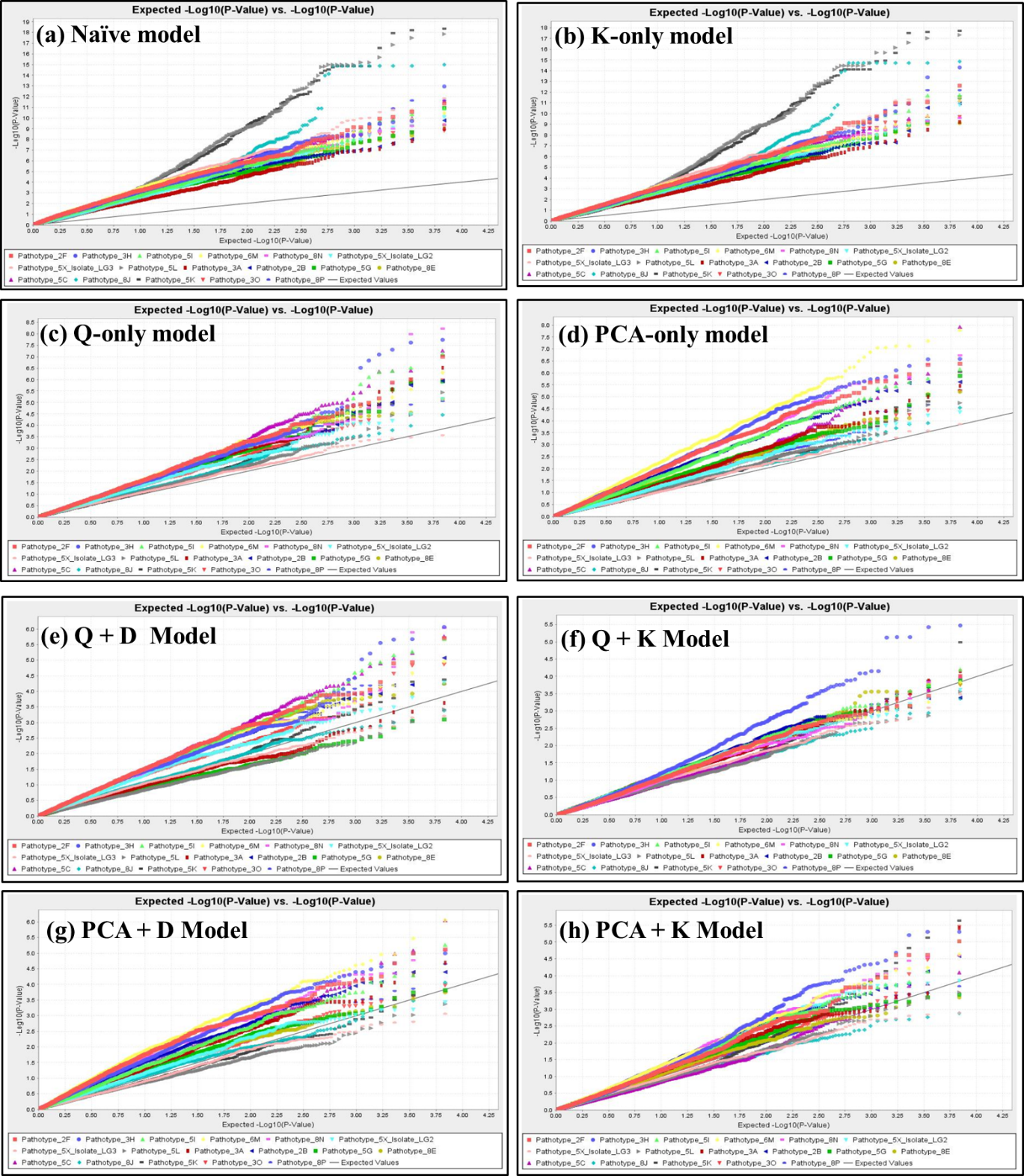


**Fig S3.** Quantile-Quantile comparison of eight GWAS models for identifying clubroot resistance loci in 124 rutabaga (*Brassica napus* ssp. *napobrassica*) accessions tested with 17 field and single-spore isolates of *Plasmodiophora brassicae* representing 16 different pathotypes. The four GLM tested comprised the Naïve(N)(a), Kinship (K)-only (b), Population structure (Q)-only (c) , and the Principal Coordinate Analysis (PCA)-only (d). The four MLM tested comprised Q + D (e), Q + K (f), PCA + D (g), PCA + K (h) models; where D is the Distance Matrix. The black line is the expected -log_10_ *P* distribution while colored lines are the observed -log_10_ *P* distribution for each of the 17 *P. brassicae* pathotypes.
